# Biomarkers of inflammation and improvement in depressive symptoms in type 1 and type 2 diabetes: differential associations with depressive symptom clusters

**DOI:** 10.1007/s00125-025-06472-w

**Published:** 2025-07-08

**Authors:** Christian Herder, Anna Zhu, Andreas Schmitt, Maria C. Spagnuolo, Bernhard Kulzer, Michael Roden, Dominic Ehrmann, Norbert Hermanns

**Affiliations:** 1 Institute for Clinical Diabetology, German Diabetes Center, Leibniz Center for Diabetes Research at Heinrich Heine University Düsseldorf, Düsseldorf, Germany; 2 German Center for Diabetes Research (DZD), München-Neuherberg, Germany; 3 Department of Endocrinology and Diabetology, Medical Faculty and University Hospital Düsseldorf, Heinrich Heine University Düsseldorf, Düsseldorf, Germany; 4 Research Institute of the Diabetes Academy Mergentheim (FIDAM), Bad Mergentheim, Germany; 5 Diabetes Center Mergentheim, Bad Mergentheim, Germany; 6 Department of Clinical Psychology and Psychotherapy, University of Bamberg, Bamberg, Germany

**Keywords:** Biomarker, CES-D, Depression, Depressive symptoms, Diabetes, Inflammation, Treatment response, Type 1 diabetes, Type 2 diabetes

## Abstract

**Aims/hypothesis:**

People with diabetes and depression show large heterogeneity in their response to depression treatment. This study aimed to identify biomarkers of subclinical inflammation that were associated with improvement of depressive symptoms in people with type 1 diabetes and type 2 diabetes.

**Methods:**

The prospective analysis combined data from three studies (DIAMOS, ECCE HOMO and DDCT). A total of 332 people with type 1 diabetes and 189 people with type 2 diabetes completed both the baseline and 1 year follow-up examinations. Depressive symptoms were assessed using the Center for Epidemiological Studies depression scale (CES-D). Associations between baseline serum levels of 76 biomarkers of inflammation and 1 year changes in depressive symptoms were estimated using multiple linear regression.

**Results:**

In people with type 2 diabetes, higher levels of 26 biomarkers were associated with greater reductions in depressive symptoms (*β*=0.128 to 0.255; *p*<0.05), whereas in people with type 1 diabetes, higher levels of 13 biomarkers were linked with lower reductions in depressive symptoms (*β*=−0.189 to −0.094; *p*<0.05). A significant effect modification was observed for 33 biomarkers (*p*_interaction_<0.05). The positive associations in type 2 diabetes were strongest for improvements in cognitive-affective and anhedonia symptoms, while the inverse associations in type 1 diabetes were strongest for improvements in somatic symptoms.

**Conclusions/interpretation:**

Higher baseline levels of multiple biomarkers of inflammation were associated with greater depression reduction in type 2 diabetes but lower depression reduction in type 1 diabetes. There were also diabetes type-specific differences in the associations with symptom clusters of depression. This suggests that different inflammation-related pathways may be relevant for the response to depression treatment in people with type 1 diabetes or type 2 diabetes.

**Graphical Abstract:**

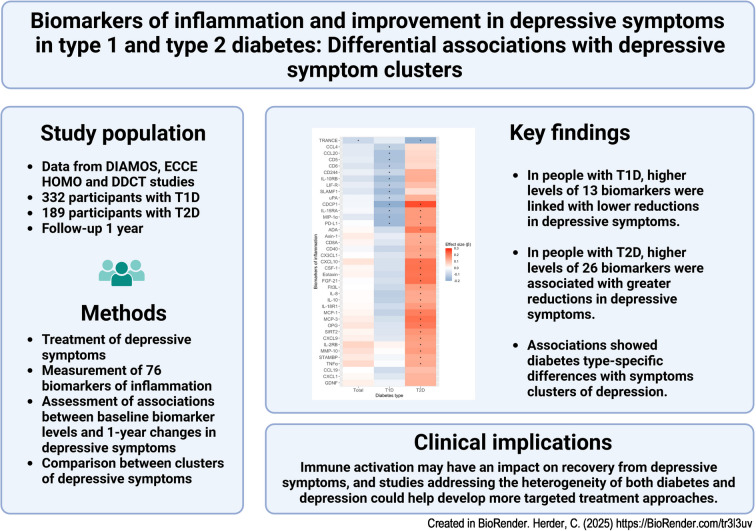

**Supplementary Information:**

The online version contains peer-reviewed but unedited supplementary material available at 10.1007/s00125-025-06472-w.


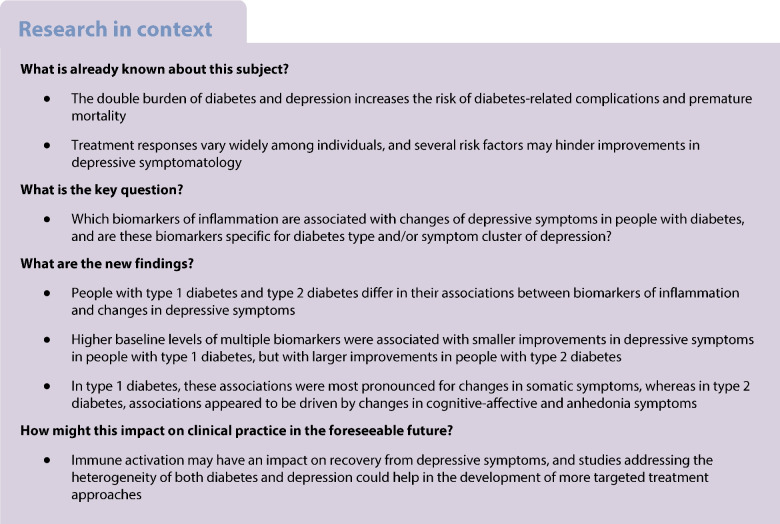


## Introduction

Depression is one of the most frequent psychosocial comorbidities in people with diabetes. The lifetime risk of major depression for people with diabetes is about twofold higher than that in the general population [1, 2]. Depression is a well-known risk factor in people with diabetes, negatively impacting diabetes self-management and quality of life [3–5]. The double burden of diabetes and depression substantially increases the risk of diabetes-related complications and mortality risk [6, 7].

Given the detrimental effects of depression in diabetes, several guidelines strongly suggest screening for depressive symptoms, with the aim of timely identification and early interventions. Interventional measures include pharmacological approaches using antidepressant drugs and non-pharmacological approaches such as cognitive behavioural therapy, or a combination of both [8]. However, treatment responses vary widely, and several risk factors may hinder change in depressive symptomatology. Such risk factors include early life adversity, greater symptom severity, chronic comorbid conditions (e.g. cardiovascular and cerebrovascular disease), coexistence of other mental disorders, substance abuse and younger age [9–11].

There is also evidence that biomarkers of inflammation may predict changes in depressive symptoms [12–14]. This is plausible because proinflammatory mechanisms contribute to the development of depression, and studies have established inflammation as a shared biological framework for both diabetes and depression [15, 16]. Furthermore, there is evidence that changes in biomarkers of inflammation are associated with changes in depressive symptoms [15]. Understanding factors associated with improvements in depressive symptoms or lack thereof is of clinical importance, and would be a step towards precision medicine.

Adding to the complexity of predicting change in depressive symptomatology is the heterogeneity of depressive symptoms. Depressive symptoms include somatic symptoms such as problems with appetite, sleep and concentration, but also cognitive-affective symptoms such as feeling down and hopeless, as well as symptoms of anhedonia such as lack of interest and joy. Thus, it is possible that changes in depressive symptoms occur in a specific symptom cluster but not in others. However, little is known about predictors of change in the various symptom clusters. Subclinical inflammation may play a role here too, as proinflammatory mechanisms have been directly linked to somatic and anhedonia symptoms of depression [17, 18].

The relevance of subclinical inflammation and its biomarkers appears of particular interest in people with diabetes and depression because both type 1 and type 2 diabetes are characterised by different types of immune activation [19–22] that may exacerbate depressive symptoms. Indeed, the association between biomarkers of inflammation and depressive symptoms may differ between type 1 diabetes and type 2 diabetes [15, 22]. However, conclusive evidence is lacking with regard to how these differential associations may also impact change in depressive symptoms.

Given the aforementioned gaps in our knowledge on predictors of changes in depressive symptoms in people with diabetes and depression, we aimed to test the hypotheses that (1) multiple biomarkers of inflammation are associated with the reduction of depressive symptoms; (2) differences in these associations exist between diabetes types; and (3) associations differ between biomarkers and changes in specific clusters of depressive symptoms (with most pronounced associations with somatic and anhedonia symptoms).

## Methods

### Study population

This longitudinal study combines data from three intervention studies comprising individuals who underwent standardised phenotyping at a specialised diabetes clinic (Diabetes Center Mergentheim, Bad Mergentheim, Germany). The studies were DIAMOS (Strengthening Diabetes Motivation [23]), ECCE HOMO (Evaluation of a Stepped Care Approach to Manage Depression in Diabetes [24]) and DDCT (Depression and Diabetes Control Trial), which are RCTs that aimed to reduce elevated depressive symptoms and diabetes distress in people with type 1 diabetes or type 2 diabetes. A detailed description of the three studies with inclusion and exclusion criteria, treatment groups and interventions is given in electronic supplementary material (ESM) Table 1. In brief, the study populations included participants with elevated depressive symptoms and/or elevated diabetes distress. As a result of this key inclusion criterion, the samples were not intended to be representative in terms of age, sex/gender, ethnicity, region, or socioeconomic background. The studies had a consistent design including pre-treatment, post-treatment and 12-month follow-up assessments, with similar treatment approaches, enabling combination of the three datasets for the present study. All individuals allocated to the treatment group received a cognitive-behavioural group treatment over five 90 min sessions (plus additional subsequent intervention steps where needed in the ECCE HOMO stepped care trial) in an inpatient setting; all control patients received diabetes care and participated in a diabetes education programme as usual at the diabetes centre. Depression outcomes were measured consistently using the Center for Epidemiological Studies depression scale (CES-D).

Each study was approved by the ethics committee of the State Medical Chamber of Baden-Württemberg, Germany (DIAMOS: 2009-034-f; ECCE HOMO: F-2013-011; DDCT: F-2015-056), and performed according to the Declaration of Helsinki. All participants provided written informed consent. The registration numbers in the ClinicalTrials.gov registry are as follows: DIAMOS: NCT01009138; ECCE HOMO: NCT01812291; DDCT: NCT02675257).

**Fig. 1:**
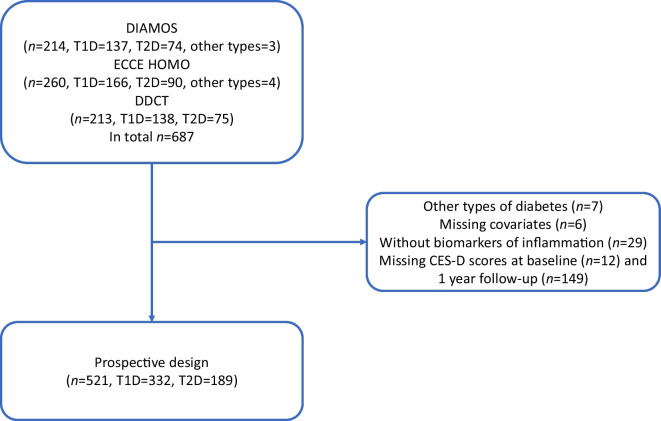
Overview of the study population, comprising participants from three intervention trials. People could fulfil more than one of the exclusion criteria. T1D, type 1 diabetes; T2D, type 2 diabetes

The present study is based on data from the baseline and 12-month follow-up examinations. The DIAMOS, ECCE HOMO and DDCT trials together enrolled 687 participants with diabetes. We excluded people with types of diabetes other than type 1 diabetes/type 2 diabetes (*n*=7), those missing covariates for statistical analysis (*n*=6), those missing data for biomarkers of inflammation (*n*=29), and those with incomplete data for depressive symptoms at baseline (*n*=12) or the 12-month follow-up (*n*=149). In total, 166 people fulfilled at least one of the exclusion criteria. Therefore, the analysis dataset consisted of data from 521 people, 332 of whom were diagnosed with type 1 diabetes and 189 of whom were diagnosed with type 2 diabetes (Fig. 1).

In a previous study, we used data from the DIAMOS, ECCE HOMO, DDCT studies and two additional samples for cross-sectional analyses on biomarkers of subclinical inflammation and depressive symptoms [22]. However, these additional samples were from studies focusing on a 17-day period using ecological momentary assessment, which did not have 12-month follow-up data, so they could not be included in the present longitudinal analysis.

### Assessment of depressive symptoms

Depressive symptoms were assessed using the German version of the CES-D [25, 26]. The CES-D consists of 20 questions requesting the frequency of various symptoms of depression within the previous week; it can be used to monitor changes in depressive symptoms over time [27, 28]. Each item is scored from 0 (‘rarely or none of the time’) to 3 (‘most or almost all the time’), with a total score of 0–60. Higher scores indicate stronger depressive symptoms. In our analyses, we used the changes in the continuous CES-D score (rather than a binary variable based on a particular cut-off) to make optimal use of the variation in symptoms.

Symptom clusters of depressive symptoms were calculated for cognitive-affective symptoms (items 3, 6, 9, 10, 14, 17, 18), somatic symptoms (items 1, 2, 5, 7, 11, 13, 20) and anhedonia symptoms (items 4, 8, 12, 16 [reversed scoring]).

### Quantification of biomarkers of inflammation

Serum levels of biomarkers of inflammation were quantified in fasting blood samples from the baseline examination that were taken between 06:30 and 08:00 hours on the working day following the day when the CES-D scale was administered.

Biomarker quantification was performed using the Olink Target 96 Inflammation assay as described previously [22]. This multimarker assay uses proximity extension assay technology and measures 92 protein biomarkers, including cytokines, chemokines, growth factors, and factors involved in acute inflammatory and immune responses, angiogenesis, fibrosis and endothelial activation. We refer to this panel as ‘biomarkers of inflammation’, but some of these biomarkers also have functions in additional pathways, reflecting the pleiotropy of most proteins in the immune system. The assay provides a relative quantification of biomarker levels in the form of normalised protein expression values, which are comparable in distribution to log_2_-transformed biomarker levels.

A full list of biomarkers with UniProt numbers and gene symbols is provided in ESM Table 2. Intra- and inter-assay CVs were calculated based on control sera measured in duplicate on each plate [22]. We defined a priori threshold levels as follows: intra-assay CV >15%, inter-assay CV >20%, and >25% of values below the detection limit. Sixteen biomarkers fulfilled at least one of these criteria (ESM Table 2), leaving 76 biomarkers for further analysis.

### Assessment of covariables

Data for covariables in regression analyses were assessed as described previously [22–24]. Demographic and diabetes-related characteristics such as age, sex, height and weight (for the calculation of BMI), diabetes type, known diabetes duration, diabetes treatment and co-medication were based on medical records or patient interviews. We also considered participation in the control group (diabetes care and diabetes education) or intervention group (additional cognitive-behavioural group treatment) in the DIAMOS, ECCE HOMO and DDCT studies as a binary covariable for which all analyses were adjusted. Information on the presence or history of diabetes-related complications was obtained in the baseline examination, and included laboratory measurements and recorded diabetes-related complications in the medical files. History of myocardial infarction, stroke or peripheral arterial occlusive disease was defined as a previous event or previous revascularisation measures. Diabetes-related chronic kidney disease was diagnosed based on an eGFR of <60 ml/min per 1.73 m^2^ and/or persistent micro-/macroalbuminuria. Diabetic retinopathy was diagnosed by an ophthalmological eye examination or based on previous laser coagulation treatment. Diabetic neuropathy was assessed using the neuropathy disability score [29].

### Statistical analysis

Baseline characteristics including serum levels of biomarkers are given as means ± SD for continuous variables and as percentages for categorical variables. Differences between diabetes types or between study cohorts were assessed using the χ^2^ test for categorical variables or the Wilcoxon rank-sum test for continuous variables. Pairwise correlations of biomarkers of inflammation were estimated based on Pearson’s correlation coefficients.

Changes in CES-D scores were calculated as the values at baseline minus the values at 1 year follow-up. To facilitate comparisons between biomarkers and between different types of depressive symptoms, baseline levels of biomarkers of inflammation and changes in the respective CES-D scores were standardised (i.e. *z*-transformed).

Associations between biomarkers of inflammation at study baseline (independent variables) and changes in CES-D scores (dependent variables) were estimated using multivariable linear regression models. Separate models were calculated for each biomarker. The results are reported as regression coefficients (*β*) and *p* values from three nested regression models adjusted for a number of covariables. Model 1 was adjusted for age, sex, study cohort, intervention/control group and baseline CES-D score. Model 2 was additionally adjusted for BMI, HbA_1c_, known diabetes duration, total cholesterol, triglycerides, use of lipid-lowering drugs (yes/no), use of non-steroidal anti-inflammatory drugs (yes/no), use of antithrombotic medication (yes/no) and use of antidepressant medication (yes/no). Model 3 was additionally adjusted for the number of diabetes-related comorbidities. All analyses were performed for the total study sample and separately for individuals with type 1 diabetes and type 2 diabetes. Additionally, differences in the associations between biomarkers and changes in the CES-D score between individuals with type 1 diabetes and type 2 diabetes were assessed by analysing the interaction between biomarkers and diabetes type.

For data visualisation, we plotted a histogram to show the distribution of Pearson correlation coefficients among biomarkers of inflammation and created a heatmap summarising associations between selected biomarkers of inflammation and changes in CES-D scores. We also plotted a chord diagram and created a heatmap to illustrate correlations between biomarkers of inflammation and baseline characteristics used as covariables in the regression models.

All analyses were performed using R software version: 4.2.2 (R Core Team, R Foundation for Statistical Computing); *p* values <0.05 were considered statistically significant.

## Results

### Study population

Baseline characteristics of the total study sample and the subgroups with type 1 diabetes and type 2 diabetes are shown in Table 1. The baseline CES-D scores were 23.6±9.6 overall (type 1 diabetes, 23.6±9.5; type 2 diabetes, 23.5±9.6), indicating clearly elevated depressive symptom levels prior to treatment, with 77.2% having elevated depressive symptoms and 57.8% having probable depression.

**Table 1:** Baseline characteristics of the study sample

Characteristic	Total	T1D	T2D	*p*
*N*	521	332	189	
Study				0.837^a^
DIAMOS	175 (33.6)	109 (32.8)	66 (34.9)	
ECCE HOMO	204 (39.2)	133 (40.1)	71 (37.6)	
DDCT	142 (27.3)	90 (27.1)	52 (27.5)	
Age (years)	46.4±13.4	40.9±12.6	56.2±8.1	<0.001
Sex, female	291 (55.9)	211 (63.6)	80 (42.3)	<0.001
BMI (kg/m^2^)	29.6±6.8	26.5±4.7	35.1±6.5	<0.001
HbA_1c_ (mmol/mol)	72.9±16.7	70.5±15.8	77.2±17.3	<0.001
HbA_1c_ (%)	8.8±1.5	8.6±1.5	9.2±1.6	<0.001
Time since diagnosis of diabetes (years)	15.3±10.4	16.7±11.6	13.0±7.4	<0.001
Total cholesterol (mmol/l)	5.10±1.29	5.13±1.02	5.10±1.63	0.880
Triglycerides (mmol/l)	1.84±1.79	1.33±1.15	2.73±2.28	<0.001
Lipid-lowering drugs	133 (25.5)	43 (13.0)	90 (47.6)	<0.001
NSAIDs	13 (2.5)	8 (2.4)	5 (2.6)	1.000
Antithrombotic drugs	104 (20.0)	28 (8.4)	76 (40.2)	<0.001
Antidepressant drugs	5 (1.0)	2 (0.6)	3 (1.6)	0.521
Number of diabetes-related comorbidities^b^	0.9±1.2	0.6±0.9	1.5±1.4	<0.001
Retinopathy	111 (21.3)	71 (21.4)	40 (21.2)	1.000
Nephropathy	49 (9.4)	18 (5.4)	31 (16.4)	<0.001
Polyneuropathy	191 (36.7)	79 (23.8)	112 (59.3)	<0.001
Diabetic foot	31 (6.0)	8 (2.4)	23 (12.2)	<0.001
PAOD	24 (4.6)	8 (2.4)	16 (8.5)	0.003
CHD	51 (9.8)	12 (3.6)	39 (20.6)	<0.001
Myocardial infarction	19 (3.6)	5 (1.5)	14 (7.4)	0.001
Stroke	15 (2.9)	3 (0.9)	12 (6.3)	0.001
CES-D				
total score	23.6±9.6	23.6±9.5	23.5±9.6	0.836
≥16 (elevated depressive symptoms)	402 (77.2)	259 (78.0)	143 (75.7)	0.539
≥22 (probable depression)	301 (57.8)	189 (56.9)	112 (59.3)	0.604

Values are means ± SD and *n* (%) for continuous and categorical variables, respectively

*p* values refer to the comparison between diabetes types

^a^*p* value for the comparison of diabetes type across the three study cohorts

^b^Diabetes-related comorbidities are retinopathy, nephropathy, polyneuropathy, diabetic foot, PAOD, CHD, myocardial infarction and stroke (maximum = 8)

NSAIDs, non-steroidal anti-inflammatory drugs; PAOD, peripheral arterial occlusive disease; T1D, type 1 diabetes; T2D, type 2 diabetes

People with type 1 diabetes were younger, included a higher proportion of women, had a lower BMI and lower HbA_1c_, longer duration of diabetes, lower serum triglycerides, less frequent use of lipid-lowering or antithrombotic drugs, and a lower total number of diabetes-related comorbidities than people with type 2 diabetes. Individuals in the two diabetes subgroups did not differ in total cholesterol levels, use of non-steroidal anti-inflammatory drugs or antidepressant drugs, or in their CES-D scores. ESM Table 3 shows the baseline characteristics stratified by the three cohorts.

There were differences between the diabetes subgroups for the majority of inflammation-related biomarkers, with higher serum levels of ten biomarkers in people with type 1 diabetes than in people with type 2 diabetes and higher serum levels of 35 biomarkers in people with type 2 diabetes than in people with type 1 diabetes (ESM Table 4). Most biomarkers showed weak or moderate positive correlations in pairwise comparisons, and inverse correlations were almost totally absent (ESM Fig. 1).

In the total study sample, biomarker levels showed multiple correlations with all covariables in the models. The highest number of correlations were observed with serum triglycerides, age, BMI, diabetes-related comorbidities and diabetes type (ESM Fig. 2). Most of these correlations were positive, as shown in the corresponding heatmap (ESM Fig. 3).

### Associations between biomarkers of inflammation and changes in depressive symptoms (CES-D total score)

CES-D depression scores decreased between baseline and the 1 year follow-up examinations in people with type 1 diabetes and type 2 diabetes by 6.8 and 5.4 points, respectively, with more pronounced reductions in the intervention groups compared with the control groups (ESM Table 5).

**Fig. 2:**
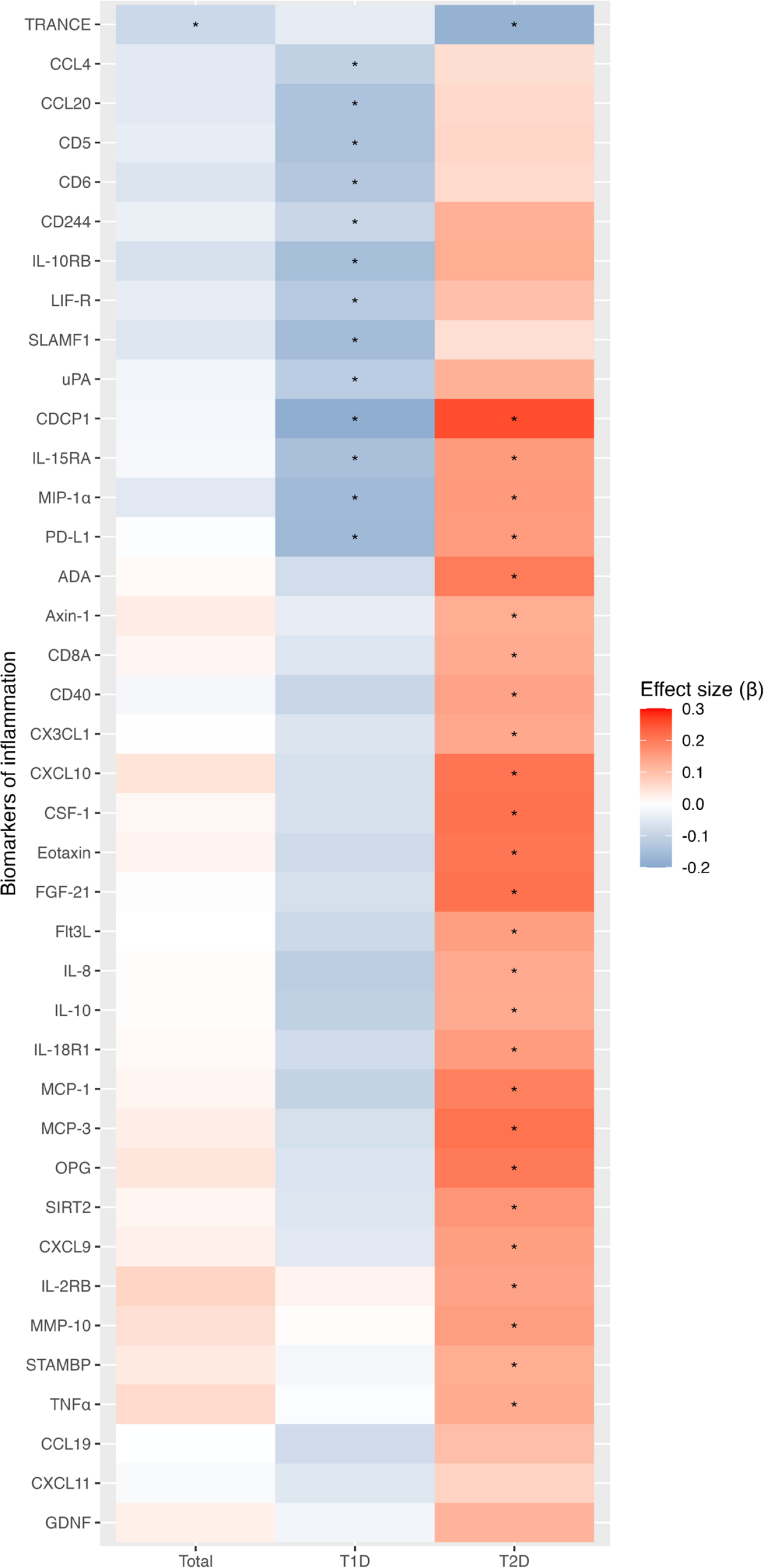
Heat map summarising associations of baseline levels of biomarkers of inflammation with changes in CES-D scores. The heatmap visualises the strength of standardised regression coefficients (*β*) for the associations between biomarkers of inflammation at baseline and changes in the CES-D score (calculated as the values at baseline minus the values at the 1 year follow-up). Higher *β* coefficients mean that higher baseline biomarker levels were associated with a higher decrease in the CES-D score. The results are from model 3 (fully adjusted); only results with *p*<0.05 for associations with changes in depressive symptoms or for interaction by diabetes type are presented. The results for all biomarkers are listed in ESM Table 8. Asterisks indicate a *p* value <0.05 for association with changes in the CES-D score. The biomarker abbreviations are defined in ESM Table 2. T1D, type 1 diabetes; T2D, type 2 diabetes

Associations between baseline levels of biomarkers of inflammation and 1 year changes in depressive symptoms were estimated in the three models of increasing complexity, with full results for models 1–3 given in ESM Tables 6–8. Figure 2 shows all biomarkers with significant findings in the fully adjusted model (model 3). The biomarker abbreviations are defined in ESM Table 2. One biomarker (TRANCE) showed an inverse association with changes in the CES-D score in the total study sample and in people with type 2 diabetes, meaning that higher TRANCE levels were associated with lower reductions in the CES-D score. In total, effect modification by diabetes type was observed for 33 biomarkers. Of those, higher levels of nine biomarkers (CCL4, CCL20, CD5, CD6, CD244, IL-10RB, LIF-R, SLAMF1, uPA) were associated with lower CES-D reductions in people with type 1 diabetes. In contrast, higher baseline levels of 17 biomarkers (ADA, axin-1, CD8A, CD40, CX3CL1, CXCL10, CSF-1, eotaxin, FGF-21, Flt3L, IL-8, IL-10, IL-18R1, MCP-1, MCP-3, OPG, SIRT2) were associated with greater reductions in depressive symptoms in people with type 2 diabetes. Four biomarkers (CDCP1, IL-15RA, MIP-1α, PD-L1) showed inverse associations with CES-D changes in people with type 1 diabetes and positive associations with CES-D changes in people with type 2 diabetes. For another three biomarkers (CCL19, CXCL1, GDNF), the interaction was significant but the associations for the subgroups were not. A final group of five biomarkers (CXCL9, IL-2RB, MMP-10, STAMBP, TNF-α) showed positive associations with changes in CES-D in people with type 2 diabetes but without significant effect modification (*p*_interaction_ ≤ 0.18). Thus, higher baseline levels of multiple biomarkers were associated with greater depressive symptom reductions in people with type 2 diabetes but with lower depressive symptom reductions in people with type 1 diabetes.

### Associations between biomarkers of inflammation and changes in clusters of depressive symptoms (CES-D sub-scales)

**Fig. 3:**
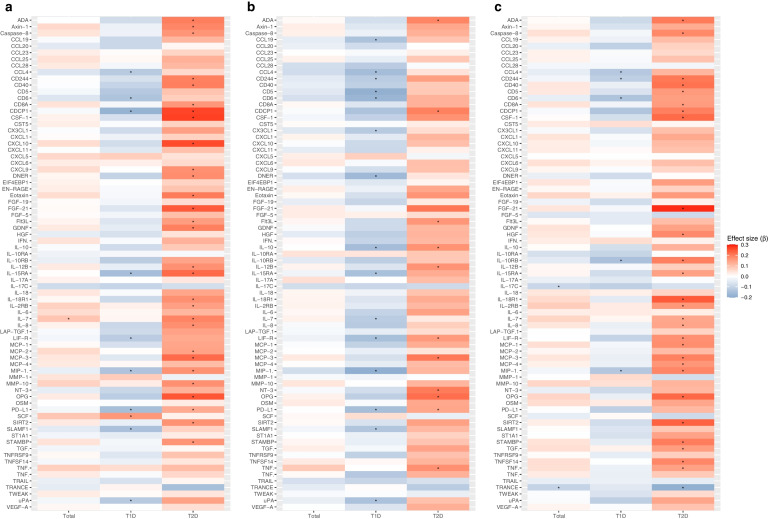
Heat map summarising associations of baseline levels of biomarkers of inflammation with changes in symptom clusters of depressive symptoms. The heatmap visualises the strength of standardised regression coefficients (*β*) for the association between biomarkers of inflammation at baseline and changes in (**a**) cognitive-affective symptoms, (**b**) somatic symptoms and (**c**) anhedonia symptoms. The results are from model 3 (fully adjusted). The results for all biomarkers are listed in ESM Table 11. Asterisks indicate a *p* value <0.05 for association with changes in depressive symptoms. The biomarker abbreviations are defined in ESM Table 2. T1D, type 1 diabetes; T2D, type 2 diabetes

Associations between baseline levels of biomarkers of inflammation and changes in cognitive-affective symptoms, somatic symptoms and anhedonia symptoms are visualised in Fig. 3 (with full results from model 3 in ESM Tables 9–11). Differences in the associations between type 1 diabetes and type 2 diabetes were observed for 30, 34 and 29 biomarkers, respectively, for cognitive-affective symptoms, somatic symptoms and anhedonia symptoms.

In people with type 2 diabetes, positive associations were most pronounced for cognitive-affective and anhedonia symptoms, with higher levels for 29 and 27 biomarkers, respectively, being associated with greater reductions in symptoms (compared with 11 biomarkers for somatic symptoms). In contrast, in people with type 1 diabetes, the inverse associations between biomarkers of inflammation were more pronounced for somatic symptoms (15 biomarkers) than for cognitive-affective symptoms (nine biomarkers) or anhedonia symptoms (five biomarkers).

## Discussion

This study shows that people with type 1 diabetes and type 2 diabetes differ in their associations between biomarkers of inflammation and changes in depressive symptoms. Higher baseline levels of multiple biomarkers were associated with smaller improvements in depressive symptoms in people with type 1 diabetes, but with larger improvements in people with type 2 diabetes. In people with type 1 diabetes, these associations were most pronounced for changes in somatic symptoms, whereas in people with type 2 diabetes, the associations appeared to be driven mainly by changes in cognitive-affective and anhedonia symptoms.

### Differential associations between biomarkers of inflammation and improvement of depressive symptoms between diabetes types

Previous studies have suggested that higher levels of several proinflammatory biomarkers such as C-reactive protein, IL-6 and TNF-α may be related to non-response to antidepressant drugs in people with major depressive disorder [13, 14]. We are not aware of any studies that (1) analysed the associations of biomarkers of inflammation with improvement of depressive symptoms upon non-pharmacological treatment; (2) were based on a comprehensive biomarker panel to characterise subclinical inflammation; or (3) addressed this topic in people with diabetes irrespective of diabetes type. Thus, our data are novel and substantially extend the current knowledge in this field.

We found associations between higher biomarker levels and smaller improvements of depressive symptoms only in people with type 1 diabetes, which is characterised by autoimmune disease activity [30]. These biomarkers included chemokines (CCL4, CCL20, MIP-1α) and soluble forms of multiple cell-surface molecules that are involved in proinflammatory signalling and activation of cells from both innate and adaptive immune systems (CD5, CD6, CD244, CDCP1, IL-10RB, IL-15RA, LIF-R, PD-L1, SLAMF1). Of note, CDCP1 is a ligand for CD6, which is expressed on certain T cells and may play a role in cell migration and chemotaxis. Higher levels of many chemokines have been found to be increased in people with depression [17, 31], but their association with treatment response remains unexplored. People with depression also show alterations in several immune cell subsets that are involved in innate and adaptive immune responses [32], but potential links to autoimmune diseases such as type 1 diabetes have not been investigated in this context. Despite these gaps in our knowledge that limit data interpretation, our study identified novel candidate biomarkers for future studies to corroborate our findings.

In contrast, higher biomarker levels were associated with stronger improvements of depressive symptoms in people with type 2 diabetes, which is characterised by subclinical inflammation [33]. These biomarkers included secreted proteins with proinflammatory activity (CSF1, Flt3L, TNF-α), chemokines (CX3CL1, CXCL10, eotaxin, IL-8, MCP-1, MCP-3, MIP-1α) and soluble forms of transmembrane proteins with functions in cell–cell communication and activation of innate and adaptive immune cells (CDCP1, CD8A, CD40, IL-2RB, IL-15RA, IL-18R1, PD-L1). This direction of association was unexpected, and we are not aware of similar findings from other studies. However, it should be noted that previous studies focused on the association between subclinical inflammation and pharmacological treatment, whereas our analysis included only studies that primarily investigated non-pharmacological interventions such as education or cognitive behavioural therapy. It would also be interesting to study the trajectories of both depressive symptoms and subclinical inflammation longitudinally to better understand our findings, but a complete set of biomarker data from the 12-month follow-up was not available in our study.

There was only a small overlap in biomarkers that were associated with improvements in depressive symptoms in opposite directions in type 1 diabetes and type 2 diabetes. One of these proteins was CDCP1, the biomarker with the highest effect size in type 2 diabetes (ESM Table 8). CDCP1 has been found to be associated with a higher risk of all-cause dementia and Alzheimer’s disease [34], but associations with other neurological or psychiatric conditions have not been reported.

So far, it is unclear why the diabetes types show differences in the associations of multiple biomarkers with changes in depressive symptoms. It is possible that they are related to the distinct types of immune activation that characterise type 1 diabetes (autoimmunity) and type 2 diabetes (subclinical inflammation) [33]. As diabetes type had no impact on the protocols and procedures in our studies, methodological or experimental issues can be excluded. It will be of great interest to compare our findings to those from people without diabetes in future studies. It is also unclear why the associations described here differ from associations that we found in our previous cross-sectional analyses to which the baseline data from the DIAMOS, ECCE HOMO and DDCT studies contributed [22]. It will be important to conduct studies that involve assessment of depressive symptoms and biomarkers at multiple timepoints to better elucidate trajectories and cause–effect relationships in the bidirectional interplay between inflammation and depression. At this stage, our observations are hypothesis-generating and may be used to design future replication studies. Comparable studies that consider the association between biomarker levels and the response to antidepressant medication are also urgently needed.

### Differential associations with diabetes symptoms clusters

Our findings indicate that the association between higher biomarker levels and lower reduction of depressive symptoms in people with type 1 diabetes was mainly driven by smaller improvements in somatic symptoms, with improvements in cognitive-affective and anhedonia symptoms being more independent of these biomarker levels at baseline. In contrast, biomarker associations with the reduction of depressive symptoms in people with type 2 diabetes were strongest for improvements in cognitive-affective and anhedonia symptoms; higher biomarker levels appeared less relevant for reduction of somatic symptoms.

Previous studies indicated that higher levels of biomarkers of inflammation were mainly associated with somatic symptoms, whereas associations were weaker for anhedonia symptoms and least pronounced for cognitive-affective symptoms [17, 18, 35–38]. However, these associations have not been compared between people with type 1 diabetes and those with type 2 diabetes.

For people with type 1 diabetes, we identified novel biomarkers that were related to a smaller improvement in depressive symptoms, which could potentially guide treatment decisions. It may be hypothesised that additional anti-inflammatory treatment may help to improve somatic symptoms in particular, in people with type 1 diabetes. The potential of anti-inflammatory drugs for the treatment of depression has been assessed in several studies [39]. It has been proposed that biomarkers of inflammation may identify endotypes of depression that would benefit from attenuating subclinical inflammation [39, 40], so studies that identify these subsets of patients are needed.

In contrast, our data for people with type 2 diabetes and high subclinical inflammation suggest that such patients are good candidates for non-pharmacological therapy approaches to reduce depressive symptoms, particularly to improve cognitive-affective and anhedonia symptoms, whereas those with lower biomarker levels may benefit more from treatment with antidepressant drugs. However, confirmation of our results in other studies is important to corroborate these hypotheses before initiating resource-intensive RCTs. Of note, the heterogeneity of diabetes is not sufficiently captured by the subdivision into type 1 diabetes and type 2 diabetes. Recent studies have suggested the existence of subtypes of type 2 diabetes, of which the severe insulin-resistant diabetes subtype is characterised by the highest inflammatory burden [15] and the highest level of depressive symptoms [41]. Thus, people with severe insulin-resistant diabetes may benefit more from psychotherapy interventions to reduce elevated depressive symptoms than those with other subtypes.

Collectively, our data suggest a role for consideration of biomarkers of inflammation in both precision diabetology and precision medicine for depression. Measurement of these and other biomarkers may be expected to lead to a better understanding of the heterogeneity of both diseases and its implications for more targeted therapies.

### Strengths and limitations

Strengths of our study include the large sample size, the individual participant data analysis from three randomised controlled trials based on similar examinations and protocols, the comprehensive biomarker phenotyping, the availability of data for both diabetes types, the analysis of symptom clusters and the adjustment for multiple confounders.

Limitations of our study mainly relate to the generalisability of the results. We analysed the reduction of depressive symptoms in the context of non-pharmacological interventions, so our results cannot be extrapolated to the response to the use of antidepressant drugs, which is most likely determined by other mechanisms and predictors. Our study sample was characterised by elevated depressive symptoms and diabetes distress, but the results may not be generalisable to people with severe major depressive disorder. Detailed data on ethnicity or ancestry were not available, so these could not be considered as potential confounders. In addition, our study cohorts mainly consisted of people of European descent, so the findings may not be generalisable to people of different ethnicity or ancestry.

### Conclusions

In our combined analysis of three intervention studies targeting depressive symptoms, higher baseline levels of multiple biomarkers of inflammation were associated with smaller improvements in depressive symptoms in people with type 1 diabetes. This finding appeared to be mainly driven by changes in somatic symptoms. In contrast, higher biomarker levels at baseline were linked with greater improvements in depressive symptoms in people with type 2 diabetes; these were related to greater reductions in cognitive-affective and anhedonia symptoms. Our findings indicate that immune activation may have an impact on recovery from depressive symptoms, and that these effects may not only differ between diabetes types but also be related to different symptom clusters of depression. If replicated by other studies, these results may help develop more targeted treatment approaches for precision medicine in diabetes and depression.

## Supplementary Information

Below is the link to the electronic supplementary material.

ESM (PDF 1385 KB)

## Data Availability

The datasets generated during and/or analysed during the current study are not publicly available due to national data protection laws, but are available from the corresponding author upon reasonable request through an individual project agreement with the Research Institute of the Diabetes Academy Mergentheim (FIDAM) and the German Diabetes Center (DDZ).

## Abbreviations

CES-D: Center for Epidemiological Studies depression scale
DDCT: Depression and Diabetes Control Trial
DIAMOS: Strengthening Diabetes Motivation
ECCE HOMO: Evaluation of a Stepped Care Approach to Manage Depression in Diabetes

## Authors’ relationships and activities

CH is Reviews Editor for *Diabetologia*. MR received honoraria from Boehringer Ingelheim and Novo Nordisk. BK received advisory board member fees from Abbott Diabetes Care, Bayer, Dexcom, Embecta and Insulet, as well as honoraria for lectures from Abbott Diabetes Care, Berlin Chemie AG, Becton Dickenson, Sanofi Germany, Roche Diabetes Care and Dexcom Germany. NH received advisory board member fees from Abbott Diabetes Care, Roche Diabetes Care and Insulet, as well as honoraria for lectures from Berlin Chemie AG, Becton Dickenson, Sanofi Germany, Roche Diabetes Care and Dexcom Germany. DE received advisory board member fees from Dexcom Germany and Roche Diabetes Care, as well as honoraria for lectures from Berlin Chemie AG, Dexcom Germany, Sanofi-Aventis Germany, Roche Diabetes Care, Eli Lilly and Boehringer Ingelheim. All other authors declare that there are no relationships or activities that might bias, or be perceived to bias, their work.
