# Supplementary material for: Biomarkers of inflammation and improvement in depressive symptoms in type 1 and type 2 diabetes: differential associations with depressive symptom clusters
Source: Diabetologia. 2025 Jul 8;68(9):2057–68. doi: 10.1007/s00125-025-06472-w (PMC12360985; doi:10.1007/s00125-025-06472-w)
Supplement: Supplementary file 1 — ESM (PDF 1385 KB) [file 125_2025_6472_MOESM1_ESM.pdf]

Electronic Supplemental Material

ESM Table 1. Overview of the three randomised controlled trials.

**Study cohort (sample size)**	**Study aim**	**Inclusion criteria**	**Exclusion criteria**	**Control group**	**Intervention group**	**Reference**
DIAMOS (Strengthening Diabetes Motivation) (*n*=214)	To evaluate a cognitive-behavioural intervention for people with subthreshold depression and diabetes distress	-Diabetes mellitus<br>-Age 18-70 years<br>-Elevated depressive symptoms (CES-D ≥16)<br>-Sufficient German language skills<br>-Written informed consent	Major depression; current schizophrenia/psychotic disorder, eating disorder, bipolar disorder, addictive disorder, or personality disorder; current use of antidepressant medication or ongoing psychotherapy; being bedridden; and under guardianship.	Standard group-based diabetes education program, consisting of five lessons (90 minutes each) that included topics such as healthy diet in diabetes, diabetes and exercise, and diabetes and legal issues.	Participantion in DIAMOS: self-management/empowerment approach delivered by certified psychologists in small groups with three to six members, comprising of five lessons of 90 min each	[1]
ECCE HOMO (Evaluation of a Stepped Care Approach to Manage Depression in Diabetes) (*n*=260)	To examine the efficacy of a stepped care approach for people with diabetes and comorbid depression and/or	-Diabetes mellitus<br>-Age 18-70 years<br>-Elevated depressive symptoms (CES-D ≥16) and/or elevated	Current severe major depression (F32.2); established diagnosis of psychotic, bipolar, personality, addictive, or severe eating disorder; current psychotherapy/antidepressant treatment; suicidal ideation; severe somatic illness (e.g.,	Diabetes care as usual at the diabetes center (education and treatment provided by the center's diabetologists, diabetes nurses and nutritionists, evaluation, adaption	-Diabetes care as usual at the diabetes center<br>-Stepped care approach/ iterative development process with up to three treatment steps: (1) diabetes-specific CBT-based group treatment for diabetes distress and depressive symptoms	[2]

	diabetes distress	diabetes-related distress (PAID ≥40)<br>-Sufficient German language skills<br>-Written informed consent	renal failure); inability to consent (based on records).	and dose-adjusting of medical treatments, structured glucose monitoring and self-management practice and educational lessons regarding acute and long-term complications, sports and activities, foods, cooking and recipes, and social aspects of diabetes.	(DIAMOS); (2) at least six weekly sessions of telephone-delivered CBT (50 minutes each; (3) in case of persistent non-response: referral for outpatient depression treatment (voluntary participation)	
DDCT (Depression and Diabetes Control Trial) (*n*=213)	To evaluate a cognitive-behavioural intervention for people with diabetes, suboptimal glycaemic control and comorbid depressive symptoms and/or diabetes distress	-Diabetes mellitus (T1D or T2D)<br>-Diabetes duration ≥1 year<br>-Age 18-70 years<br>-HbA1c >7.5% (58 mmol/mol)<br>-Elevated depressive symptoms (CES-D ≥16) and/or elevated diabetes	Severe major depressive disorder, current psychiatric and/or psychotherapeutic treatment, current antidepressive medical treatment, suicidal ideation. acute mental disorder (schizophrenia or other psychotic disorder, bipolar disorder, severe eating disorder, substance use disorder), history of personality disorder, severe somatic illnesses (dialysis-dependent nephropathy, acute cancer, severe heart disease, severe neurologic illness, severe autoimmune disease, terminal illness, bedriddenness, guardianship.	Treatment-as-usual (standard diabetes education; see ECCE HOMO)	-Treatment-as-usual (standard diabetes education)<br>-Five group sessions of diabetes-specific cognitive-behavioural group treatment for diabetes patients with depressive symptoms and/or diabetes distress and suboptimal glycaemic control	[3]

		distress (PAID ≥ 40)<br>-Sufficient German language skills<br>-Written informed consent				

CBT, cognitive behavioural therapy; PAID, Problem Areas In Diabetes Scale.

References:

1. Hermanns N, Schmitt A, Gahr A, Herder C, Nowotny B, Roden M, Ohmann C, Kruse J, Haak T, Kulzer B. The effect of a Diabetes-Specific Cognitive Behavioral Treatment Program (DIAMOS) for patients with diabetes and subclinical depression: results of a randomized controlled trial. Diabetes Care. 2015 Apr;38(4):551-60. doi: 10.2337/dc14-1416.

2. Schmitt A, Kulzer B, Reimer A, Herder C, Roden M, Haak T, Hermanns N. Evaluation of a Stepped Care Approach to Manage Depression and Diabetes Distress in Patients with Type 1 Diabetes and Type 2 Diabetes: Results of a Randomized Controlled Trial (ECCE HOMO Study). Psychother Psychosom. 2022;91(2):107-122. doi: 10.1159/000520319.

3. Schmitt A, Kulzer B, Reimer A, Haak T, Hermanns N. Depression and Diabetes Control Trial (DDCT): Evaluation einer kognitiv-behavioralen Behandlung für Menschen mit Diabetes mit erhöhten affektiven Belastungen und suboptimaler Glukoseeinstellung. Diabetologie und Stoffwechsel 2019;14(S 01): S25-S26. doi: 10.1055/s-0039-1688179.

ESM Table 2. Biomarkers in the OLINK Inflammation panel.

Biomarker	Full name	UniProt No	Gene symbol	Excluded
ADA	Adenosine deaminase	P00813	*ADA*	
ARTN*	Artemin	Q5T4W7	*ARTN*	x
AXIN1	Axis inhibition protein 1	O15169	*AXIN1*	
Beta-NGF*	β-nerve growth factor	P01138	*NGF*	x
CASP-8	Caspase-8	Q14790	*CASP8*	
CCL3	C-C motif chemokine 3	P10147	*CCL3*	
CCL4	C-C motif chemokine 4	P13236	*CCL4*	
CCL19	C-C motif chemokine 19	Q99731	*CCL19*	
CCL20	C-C motif chemokine 20	P78556	*CCL20*	
CCL23	C-C motif chemokine 23	P55773	*CCL23*	
CCL25	C-C motif chemokine 25	O15444	*CCL25*	
CCL28	C-C motif chemokine 28	Q9NRJ3	*CCL28*	
CD5	T-cell surface glycoprotein CD5	P06127	*CD5*	
CD6	T-cell differentiation antigen CD6	P30203	*CD6*	
CD8A	T-cell surface glycoprotein CD8α chain	P01732	*CD8A*	
CD40	Tumor necrosis factor receptor superfamily member 5	P25942	*CD40*	
CD244	Natural killer cell receptor 2B4	Q9BZW8	*CD244*	
CDCP1	CUB domain-containing protein 1	Q9H5V8	*CDCP1*	
CSF-1	Macrophage colony-stimulating factor 1	P09603	*CSF1*	
CST5	Cystatin-D	P28325	*CST5*	
CX3CL1	Fractalkine	P78423	*CX3CL1*	
CXCL1	Growth-regulated α protein	P09341	*CXCL1*	
CXCL5	C-X-C motif chemokine 5	P42830	*CXCL5*	
CXCL6	C-X-C motif chemokine 6	P80162	*CXCL6*	
CXCL9	C-X-C motif chemokine 9	Q07325	*CXCL9*	
CXCL10	C-X-C motif chemokine 10	P02778	*CXCL10*	
CXCL11	C-X-C motif chemokine 11	O14625	*CXCL11*	
DNER	Delta and Notch-like epidermal growth factor-related receptor	Q8NFT8	*DNER*	
EIF4EBP1	Eukaryotic translation initiation factor 4E-binding protein 1	Q13541	*EIF4EBP1*	
EN-RAGE	Protein S100-A12 (EN-RAGE)	P80511	*S100A12*	
Eotaxin	Eotaxin (CCL11)	P51671	*CCL11*	
FGF-5	Fibroblast growth factor 5	P12034	*FGF5*	
FGF-19	Fibroblast growth factor 19	O95750	*FGF19*	
FGF-21	Fibroblast growth factor 21	Q9NSA1	*FGF21*	
FGF-23*	Fibroblast growth factor 23	Q9GZV9	*FGF23*	x
Flt3L	Fms-related tyrosine kinase 3 ligand	P49771	*FLT3LG*	
GDNF	Glial cell line-derived neurotrophic factor	P39905	*GDNF*	
HGF	Hepatocyte growth factor	P14210	*HGF*	
IFNγ	Interferon-γ	P01579	*IFNG*	

IL-1α*	Interleukin-1α	P01583	*IL1A*	x
IL-2	Interleukin-2	P60568	*IL2*	
IL-2RB	Interleukin-2 receptor subunit β	P14784	*IL2RB*	
IL-4*	Interleukin-4	P05112	*IL4*	x
IL-5*	Interleukin-5	P05113	*IL5*	x
IL-6	Interleukin-6	P05231	*IL6*	
IL-7	Interleukin-7	P13232	*IL7*	
IL-8	Interleukin-8	P10145	*CXCL8*	
IL-10	Interleukin-10	P22301	*IL10*	
IL-10RA	Interleukin-10 receptor subunit α	Q13651	*IL10RA*	
IL-10RB	Interleukin-10 receptor subunit β	Q08334	*IL10RB*	
IL-12B	Interleukin-12 subunit β	P29460	*IL12B*	
IL-13*	Interleukin-13	P35225	*IL13*	x
IL-15RA	Interleukin-15 receptor subunit α	Q13261	*IL15RA*	
IL-17A	Interleukin-17A	Q16552	*IL17A*	
IL-17C	Interleukin-17C	Q9P0M4	*IL17C*	
IL-18	Interleukin-18	Q14116	*IL18*	
IL-18R1	Interleukin-18 receptor 1	Q13478	*IL18R1*	
IL-20*	Interleukin-20	Q9NYY1	*IL20*	x
IL-20RA*	Interleukin-20 receptor subunit α	Q9UHF4	*IL20RA*	x
IL-22 RA1*	Interleukin-22 receptor subunit α1	Q8N6P7	*IL22RA1*	x
IL-24*	Interleukin-24	Q13007	*IL24*	x
IL-33*	Interleukin-33	O95760	*IL33*	x
LAP TGFβ1	Latency-associated peptide transforming growth factor β1	P01137	*TGFB1*	
LIF*	Leukemia inhibitory factor	P15018	*LIF*	x
LIF-R	Leukemia inhibitory factor receptor	P42702	*LIFR*	
MCP-1	Monocyte chemotactic protein 1 (CCL2)	P13500	*CCL2*	
MCP-2	Monocyte chemotactic protein 2 (MCP-2, CCL8)	P80075	*CCL8*	
MCP-3	Monocyte chemotactic protein 3 (CCL7)	P80098	*CCL7*	
MCP-4	Monocyte chemotactic protein 4 (CCL13)	Q99616	*CCL13*	
MMP-1	Matrix metalloproteinase-1	P03956	*MMP1*	
MMP-10	Matrix metalloproteinase-10 (SL-2)	P09238	*MMP10*	
Neurturin*	Neurturin	Q99748	*NRTN*	x
NT-3	Neurotrophin-3	P20783	*NTF3*	
OPG	Osteoprotegerin	O00300	*TNFRSF11B*	
OSM	Oncostatin-M	P13725	*OSM*	
PD-L1	Programmed cell death 1 ligand 1	Q9NZQ7	*CD274*	
SCF	Stem cell factor (c-Kit-ligand)	P21583	*KITLG*	
SIRT2	SIR2-like protein 2	Q8IXJ6	*SIRT2*	
SLAMF1	Signaling lymphocytic activation molecule (SLAM)	Q13291	*SLAMF1*	
ST1A1	Sulfotransferase 1A1	P50225	*SULT1A1*	
STAMBP	STAM-binding protein	O95630	*STAMBP*	
TGFα	Transforming growth factor α	P01135	*TGFA*	

TNFα	Tumor necrosis factor-α	P01375	*TNF*	
TNFβ	Tumor necrosis factor-β (lymphotoxin-α/LT-α)	P01374	*LTA*	
TNFRSF9	Tumor necrosis factor receptor superfamily member 9	Q07011	*TNFRSF9*	
TNFSF14	Tumor necrosis factor ligand superfamily member 14 (LIGHT)	O43557	*TNFSF14*	
TRAIL	TNF-related apoptosis-inducing ligand (TNFSF10)	P50591	*TNFSF10*	
TRANCE	TNF-related activation-induced cytokine (TRANCE, TNFSF11, RANKL, OPGL)	O14788	*TNFSF11*	
TSLP*	Thymic stromal lymphopoietin	Q969D9	*TSLP*	x
TWEAK	Tumor necrosis factor (Ligand) superfamily, member 12 (TWEAK)	O43508	*TNFSF12*	
uPA	Urokinase-type plasminogen activator	P00749	*PLAU*	
VEGF-A	Vascular endothelial growth factor A	P15692	*VEGFA*	

*Excluded from analysis because intra-assay CV >15%, interassay-CV >20% or >25% values below the limit of detection.

ESM Table 3. Baseline characteristics by cohort.

**Characteristics**	**Study**			
	**DIAMOS**	**ECCE HOMO**	**DDCT**	***p***
n	175	204	142	
Age (years)	45.2 ± 13.4	46.6 ± 13.5	47.6 ± 13.0	0.272
Sex , females (%)	98 (56.0)	111 (54.4)	82 (57.7)	0.827
Diabetes type				0.837
Type 1 diabetes	109 (62.3)	133 (65.2)	90 (63.4)	
Type 2 diabetes	66 (37.7)	71 (34.8)	52 (36.6)	
Body mass index (kg/m²)	29.6 ± 7.1	29.7 ± 6.7	29.6 ± 6.6	0.990
HbA1c (%)	8.8 ± 1.7	8.6 ± 1.5	9.2 ± 1.3	0.006
HbA1c (mmol/mol)	72.3 ± 18.4	70.9 ± 16.6	76.6 ± 13.9	0.006
Time since diagnosis of diabetes (years)	14.3 ± 10.7	15.4 ± 10.6	16.5 ± 9.7	0.179
Total cholesterol (mmol/l)	5.26 ± 1.63	4.92 ± 0.93	5.21 ± 1.17	0.017
Triglycerides (mmol/l)	1.95 ± 2.24	1.71 ± 1.58	1.88 ± 1.37	0.378
Lipid-lowering drugs (%)	52 (29.7)	46 (22.5)	35 (24.6)	0.269
NSAIDs (%)	3 (1.7)	8 (3.9)	2 (1.4)	0.242
Antithrombotic drugs (%)	41 (23.4)	34 (16.7)	29 (20.4)	0.256
Antidepressant drugs (%)	4 (2.3)	0	1 (0.7)	0.070
Number of diabetes-related comorbidities*	1.0 ± 1.3	0.7 ± 1.1	1.2 ± 1.3	0.002
Retinopathy (%)	46 (26.3)	41 (20.1)	24 (16.9)	0.110
Nephropathy (%)	19 (10.9)	14 (6.9)	16 (11.3)	0.278
Polyneuropathy (%)	66 (37.7)	53 (26.0)	72 (50.7)	<0.001
Diabetic foot (%)	5 (2.9)	9 (4.4)	17 (12.0)	0.001
PAOD (%)	11 (6.3)	7 (3.4)	6 (4.2)	0.404
Coronary heart disease (%)	16 (9.1)	15 (7.4)	20 (14.1)	0.110
Myocardial infarction (%)	7 (4.0)	3 (1.5)	9 (6.3)	0.057

Stroke (%)	6 (3.4)	6 (2.9)	3 (2.1)	0.783
CES-D	23.2 ± 8.1	23.7 ± 9.5	23.8 ± 11.3	0.845

Data are given as mean ± SD and *n* (%) for continuous and categorical variables, respectively.

*Diabetes-related comorbidities include retinopathy, nephropathy, polyneuropathy, diabetic foot, PAOD, coronary heart disease, myocardial infarction and stroke (max. 8).

NSAIDs, non-steroidal anti-inflammatory drugs; PAOD, peripheral arterial occlusive disease; T1D, type 1 diabetes; T2D, type 2 diabetes.

ESM Table 4. Baseline serum levels of biomarkers of inflammation in the study sample (total and stratified by diabetes type).

Biomarkers	Total	T1D	T2D	*p*
ADA	5.37 ± 0.48	5.38 ± 0.50	5.36 ± 0.44	0.639
Axin-1	2.54 ± 0.64	2.56 ± 0.62	2.50 ± 0.68	0.338
Caspase-8	4.00 ± 0.53	3.96 ± 0.53	4.05 ± 0.53	0.056
CCL4	8.18 ± 0.60	8.12 ± 0.59	8.29 ± 0.60	**0.001**
CCL19	9.53 ± 0.71	9.39 ± 0.64	9.78 ± 0.76	**<0.001**
CCL20	7.67 ± 1.08	7.53 ± 0.99	7.93 ± 1.17	**<0.001**
CCL23	11.07 ± 0.45	11.07 ± 0.44	11.08 ± 0.47	0.792
CCL25	7.16 ± 0.67	7.15 ± 0.68	7.17 ± 0.64	0.782
CCL28	2.63 ± 0.46	2.67 ± 0.47	2.57 ± 0.43	**0.013**
CD5	6.60 ± 0.42	6.57 ± 0.38	6.67 ± 0.48	**0.005**
CD6	5.84 ± 0.51	5.84 ± 0.51	5.85 ± 0.51	0.864
CD8A	10.35 ± 0.64	10.28 ± 0.62	10.47 ± 0.67	**0.001**
CD40	11.75 ± 0.39	11.72 ± 0.39	11.80 ± 0.38	**0.014**
CD244	6.55 ± 0.39	6.55 ± 0.42	6.55 ± 0.34	0.939
CDCP1	3.05 ± 0.75	2.79 ± 0.67	3.49 ± 0.68	**<0.001**
CSF-1	10.36 ± 0.24	10.32 ± 0.24	10.44 ± 0.23	**<0.001**
CST5	5.75 ± 0.56	5.69 ± 0.55	5.85 ± 0.56	**0.002**
CX3CL1	4.33 ± 0.45	4.38 ± 0.47	4.26 ± 0.42	**0.005**
CXCL1	10.12 ± 0.51	10.10 ± 0.49	10.15 ± 0.53	0.311
CXCL5	12.35 ± 0.63	12.37 ± 0.60	12.31 ± 0.67	0.275
CXCL6	10.33 ± 0.59	10.28 ± 0.58	10.41 ± 0.61	**0.010**
CXCL9	7.22 ± 0.80	7.13 ± 0.78	7.40 ± 0.81	**<0.001**
CXCL10	9.95 ± 0.84	9.86 ± 0.80	10.11 ± 0.88	**0.001**
CXCL11	8.56 ± 0.79	8.52 ± 0.81	8.64 ± 0.76	0.071
DNER	9.07 ± 0.29	9.15 ± 0.26	8.94 ± 0.29	**<0.001**
EIF4EBP1	7.39 ± 0.99	7.25 ± 1.02	7.63 ± 0.89	**<0.001**
EN-RAGE	5.56 ± 0.91	5.58 ± 0.91	5.52 ± 0.90	0.419
Eotaxin	9.02 ± 0.49	8.98 ± 0.52	9.11 ± 0.43	**0.003**
FGF-5	2.30 ± 0.38	2.30 ± 0.41	2.29 ± 0.32	0.863
FGF-19	8.60 ± 0.94	8.87 ± 0.84	8.15 ± 0.93	**<0.001**
FGF-21	5.73 ± 1.67	5.03 ± 1.43	6.96 ± 1.30	**<0.001**
Flt3L	9.35 ± 0.44	9.29 ± 0.43	9.47 ± 0.43	**<0.001**
GDNF	1.85 ± 0.45	1.82 ± 0.47	1.90 ± 0.42	**0.043**
HGF	10.28 ± 0.51	10.16 ± 0.51	10.48 ± 0.46	**<0.001**
IFNγ	6.92 ± 1.10	6.83 ± 1.00	7.09 ± 1.23	**0.008**
IL-2RB	2.10 ± 0.48	2.09 ± 0.47	2.12 ± 0.50	0.590
IL-6	3.90 ± 0.95	3.60 ± 0.83	4.41 ± 0.91	**<0.001**
IL-7	4.17 ± 0.51	4.18 ± 0.50	4.16 ± 0.54	0.679
IL-8	6.35 ± 0.71	6.22 ± 0.65	6.58 ± 0.74	**<0.001**
IL-10	3.94 ± 0.62	3.89 ± 0.56	4.05 ± 0.71	0.003
IL-10RA	1.89 ± 0.64	1.91 ± 0.67	1.86 ± 0.60	0.353
IL-10RB	7.64 ± 0.33	7.58 ± 0.33	7.74 ± 0.31	**<0.001**
IL-12B	7.58 ± 0.63	7.54 ± 0.61	7.67 ± 0.65	**0.015**
IL-15RA	2.22 ± 0.31	2.17 ± 0.29	2.29 ± 0.33	**<0.001**
IL-17A	2.49 ± 0.63	2.46 ± 0.60	2.53 ± 0.69	0.202
IL-17C	2.95 ± 0.73	2.93 ± 0.74	2.97 ± 0.71	0.608
IL-18	9.61 ± 0.57	9.52 ± 0.54	9.77 ± 0.59	**<0.001**
IL-18R1	8.63 ± 0.49	8.51 ± 0.45	8.84 ± 0.50	**<0.001**

LAP TGFβ1	9.19 ± 0.43	9.18 ± 0.46	9.19 ± 0.36	0.895
LIF-R	4.05 ± 0.30	4.04 ± 0.30	4.08 ± 0.30	0.172
MCP-1	13.35 ± 0.50	13.28 ± 0.48	13.46 ± 0.51	**<0.001**
MCP-2	10.66 ± 0.68	10.65 ± 0.66	10.67 ± 0.70	0.687
MCP-3	2.43 ± 0.63	2.24 ± 0.57	2.74 ± 0.61	**<0.001**
MCP-4	15.22 ± 0.66	15.13 ± 0.67	15.37 ± 0.62	**<0.001**
MIP-1α	7.63 ± 0.60	7.51 ± 0.58	7.85 ± 0.57	**<0.001**
MMP-1	15.47 ± 0.72	15.43 ± 0.68	15.52 ± 0.78	0.180
MMP-10	9.44 ± 0.65	9.40 ± 0.67	9.51 ± 0.60	**0.049**
NT-3	2.80 ± 0.41	2.88 ± 0.42	2.67 ± 0.34	**<0.001**
OPG	10.43 ± 0.41	10.38 ± 0.38	10.52 ± 0.43	**<0.001**
OSM	6.72 ± 0.82	6.67 ± 0.82	6.80 ± 0.82	0.070
PD-L1	5.75 ± 0.39	5.72 ± 0.36	5.79 ± 0.44	**0.048**
SCF	9.91 ± 0.46	9.99 ± 0.40	9.78 ± 0.52	**<0.001**
SIRT2	3.44 ± 0.66	3.46 ± 0.67	3.42 ± 0.64	0.462
SLAMF1	2.91 ± 0.44	2.82 ± 0.40	3.06 ± 0.46	**<0.001**
ST1A1	3.38 ± 1.00	3.45 ± 1.01	3.25 ± 0.97	**0.023**
STAMBP	4.02 ± 0.51	4.00 ± 0.51	4.05 ± 0.50	0.267
TGFα	4.72 ± 0.72	4.73 ± 0.72	4.71 ± 0.72	0.747
TNFRSF9	6.58 ± 0.52	6.56 ± 0.47	6.62 ± 0.59	0.213
TNFSF14	7.20 ± 0.70	7.23 ± 0.71	7.14 ± 0.68	0.166
TNFα	4.14 ± 0.49	4.05 ± 0.43	4.30 ± 0.54	**<0.001**
TNFβ	5.23 ± 0.47	5.27 ± 0.42	5.17 ± 0.54	**0.021**
TRAIL	7.96 ± 0.32	7.96 ± 0.34	7.97 ± 0.30	0.867
TRANCE	5.30 ± 0.64	5.35 ± 0.64	5.20 ± 0.63	**0.008**
TWEAK	9.50 ± 0.41	9.61 ± 0.37	9.31 ± 0.39	**<0.001**
uPA	10.38 ± 0.35	10.37 ± 0.36	10.39 ± 0.34	0.553
VEGF-A	12.16 ± 0.56	12.09 ± 0.56	12.28 ± 0.55	**<0.001**

Data are given as mean ± SD of normalised protein expression (NPX) values.

ESM Table 5. CES-D scores at baseline and 1-year follow-up.

Study cohort	Group	Baseline			1-year follow-up		
		**Total**	**T1D**	**T2D**	**Total**	**T1D**	**T2D**
All	Combined	23.6 ± 9.6	23.6 ± 9.5	23.5 ± 9.6	17.3 ± 10.9	16.8 ± 11.1	18.1 ± 10.5
	Intervention	24.0 ± 9.4	24.6 ± 9.6	23.1 ± 9.1	16.4 ± 10.4	16.0 ± 10.5	17.0 ± 10.3
	Control	23.1 ± 9.7	22.8 ± 9.4	23.9 ± 10.4	18.2 ± 11.3	17.5 ± 11.6	19.7 ± 10.7
DIAMOS	Combined	23.2 ± 8.1	24.1 ± 8.4	21.9 ± 7.4	18.3 ± 11.1	18.1 ± 11.9	18.6 ± 9.7
	Intervention	24.3 ± 7.4	26.6 ± 7.2	21.6 ± 6.9	16.9 ± 10.2	17.4 ± 11.4	16.4 ± 8.7
	Control	22.2 ± 8.6	22.0 ± 8.8	22.4 ± 8.2	19.6 ± 11.8	18.6 ± 12.4	22.0 ± 10.3
ECCE HOMO	Combined	23.7 ± 9.5	23.3 ± 9.3	24.5 ± 9.8	15.7 ± 10.1	15.2 ± 10.3	16.7 ± 9.6
	Intervention	24.1 ± 9.1	24.1 ± 9.4	24.1 ± 8.6	14.7 ± 9.6	14.5 ± 9.5	15.2 ± 10.0
	Control	23.3 ± 9.8	22.4 ± 9.2	24.9 ± 10.9	16.6 ± 10.5	15.8 ± 11.1	18.0 ± 9.1
DDCT	Combined	23.8 ± 11.3	23.7 ± 11.1	24.0 ± 11.7	18.4 ± 11.5	17.7 ± 10.9	19.5 ± 12.5
	Intervention	23.5 ± 11.6	23.1 ± 11.8	24.0 ± 11.4	18.1 ± 11.4	17.0 ± 10.9	19.4 ± 11.9
	Control	24.2 ± 11.0	24.2 ± 10.5	24.0 ± 12.6	18.7 ± 11.8	18.4 ± 11.0	19.8 ± 14.1

Data are given as mean ± SD.

ESM Table 6. Associations between biomarkers of inflammation and changes in depressive symptoms (model 1).

**Biomarkers**	**Total**		**T1D**		**T2D**		$p_{interaction}$
	**β**	***p***	**β**	***p***	**β**	***p***	
ADA	0.004	0.911	-0.069	0.160	**0.168**	**0.012**	**0.008**
Axin-1	0.030	0.436	-0.041	0.432	**0.126**	**0.034**	**0.036**
Caspase-8	0.020	0.622	-0.040	0.441	0.118	0.064	0.075
CCL4	-0.051	0.185	**-0.117**	**0.019**	0.062	0.324	**0.029**
CCL19	-0.016	0.683	-0.088	0.104	0.103	0.085	**0.027**
CCL20	-0.054	0.165	**-0.141**	**0.009**	0.066	0.252	**0.013**
CCL23	0.011	0.782	-0.029	0.577	0.079	0.169	0.232
CCL25	0.003	0.937	-0.042	0.397	0.093	0.149	0.163
CCL28	-0.0009	0.982	-0.016	0.744	0.022	0.733	0.697
CD5	-0.056	0.149	**-0.146**	**0.008**	0.051	0.358	**0.012**
CD6	-0.072	0.058	**-0.135**	**0.006**	0.042	0.507	**0.024**
CD8A	0.002	0.949	-0.064	0.222	0.115	0.051	**0.020**
CD40	-0.020	0.619	-0.091	0.073	0.111	0.081	**0.030**
CD244	-0.038	0.318	-0.090	0.051	0.119	0.099	**0.018**
CDCP1	-0.042	0.338	**-0.199**	**0.001**	**0.212**	**0.002**	**<0.001**
CSF-1	-0.007	0.860	-0.084	0.099	**0.173**	**0.011**	**0.005**
CST5	0.022	0.568	-0.001	0.981	0.058	0.344	0.630
CX3CL1	0.007	0.862	-0.050	0.303	0.124	0.060	0.058
CXCL1	-0.000	0.998	-0.066	0.204	0.110	0.069	**0.036**
CXCL5	0.004	0.917	0.002	0.971	0.009	0.891	0.806
CXCL6	0.004	0.917	-0.023	0.663	0.063	0.303	0.280
CXCL9	0.020	0.616	-0.047	0.385	**0.142**	**0.025**	0.083
CXCL10	0.044	0.269	-0.063	0.241	**0.198**	**0.001**	**0.003**
CXCL11	-0.014	0.717	-0.055	0.271	0.069	0.273	0.157
DNER	-0.007	0.854	-0.068	0.225	0.047	0.458	0.158
EIF4EBP1	0.002	0.956	-0.034	0.491	0.089	0.200	0.222
EN-RAGE	-0.009	0.819	-0.046	0.364	0.060	0.343	0.205
Eotaxin	0.019	0.645	-0.078	0.143	**0.194**	**0.005**	**0.005**
FGF-5	-0.050	0.228	-0.066	0.165	0.003	0.972	0.606
FGF-19	-0.020	0.611	-0.060	0.276	0.022	0.721	0.322
FGF-21	-0.032	0.437	-0.098	0.085	**0.165**	**0.048**	**0.024**
Flt3L	-0.010	0.809	-0.092	0.084	**0.135**	**0.037**	**0.024**
GDNF	0.025	0.523	-0.012	0.810	0.106	0.116	0.212
HGF	-0.042	0.279	-0.095	0.059	0.073	0.283	0.055
IFNγ	0.021	0.598	-0.058	0.302	**0.110**	**0.042**	0.060
IL-2RB	0.062	0.104	0.021	0.678	**0.131**	**0.027**	0.173
IL-6	0.004	0.919	-0.067	0.281	0.092	0.167	0.112
IL-7	0.049	0.201	-0.009	0.861	**0.126**	**0.026**	0.081

IL-8	-0.005	0.902	**-0.118**	**0.037**	**0.134**	**0.021**	**0.004**
IL-10	0.001	0.979	-0.105	0.057	**0.122**	**0.023**	**0.004**
IL-10RA	-0.017	0.660	-0.027	0.572	-0.001	0.993	0.748
IL-10RB	-0.079	0.043	**-0.159**	**0.001**	0.095	0.155	**0.004**
IL-12B	-0.0002	0.997	-0.033	0.537	0.067	0.285	0.316
IL-15RA	-0.029	0.453	**-0.141**	**0.010**	**0.130**	**0.027**	**0.003**
IL-17A	-0.004	0.915	-0.031	0.556	0.026	0.645	0.406
IL-17C	-0.073	0.057	-0.092	0.061	-0.051	0.427	0.598
IL-18	-0.010	0.792	-0.068	0.203	0.095	0.116	0.066
IL-18R1	-0.013	0.742	-0.094	0.088	0.116	0.059	**0.014**
LAP TGFβ1	-0.014	0.720	-0.039	0.406	0.058	0.413	0.223
LIF-R	-0.044	0.264	**-0.110**	**0.029**	0.073	0.247	**0.043**
MCP-1	0.011	0.789	**-0.111**	**0.037**	**0.194**	**0.001**	**<0.001**
MCP-2	0.016	0.666	-0.039	0.445	0.102	0.082	0.072
MCP-3	0.008	0.840	-0.091	0.101	**0.194**	**0.003**	**0.003**
MCP-4	0.006	0.876	-0.043	0.398	0.105	0.118	0.134
MIP-1α	-0.062	0.121	**-0.171**	**0.001**	**0.138**	**0.035**	**0.001**
MMP-1	-0.015	0.670	0.004	0.935	-0.044	0.449	0.539
MMP-10	0.039	0.314	-0.000	0.999	**0.146**	**0.029**	0.131
NT-3	-0.029	0.456	-0.072	0.130	0.077	0.313	0.074
OPG	0.031	0.451	-0.060	0.289	**0.172**	**0.006**	**0.032**
OSM	0.014	0.720	-0.026	0.612	0.092	0.138	0.118
PD-L1	-0.010	0.796	**-0.150**	**0.006**	**0.148**	**0.006**	**<0.001**
SCF	0.038	0.328	0.076	0.185	-0.007	0.899	0.276
SIRT2	0.010	0.789	-0.064	0.193	**0.153**	**0.016**	**0.008**
SLAMF1	-0.067	0.093	**-0.151**	**0.006**	0.048	0.414	**0.021**
ST1A1	-0.005	0.898	-0.057	0.249	0.091	0.155	0.057
STAMBP	0.028	0.477	-0.026	0.600	0.122	0.050	0.076
TGFα	-0.000	0.998	-0.034	0.494	0.067	0.281	0.189
TNFRSF9	-0.031	0.423	-0.088	0.111	0.038	0.492	0.145
TNFSF14	0.006	0.869	-0.037	0.449	0.087	0.175	0.100
TNFα	0.044	0.258	-0.020	0.729	**0.121**	**0.027**	0.100
TNFβ	0.044	0.265	0.020	0.738	0.064	0.247	0.480
TRAIL	-0.051	0.187	-0.095	0.053	0.023	0.721	0.157
TRANCE	-0.086	0.029	-0.053	0.299	**-0.153**	**0.017**	0.350
TWEAK	0.012	0.757	0.018	0.740	-0.028	0.658	0.715
uPA	-0.016	0.686	-0.099	0.051	0.125	0.051	**0.014**
VEGF-A	-0.016	0.676	-0.055	0.271	0.068	0.298	0.155

Results are from model 1 adjusted for age, sex, study cohort, intervention/control group and baseline CES-D score. $p_{interaction}$ refers to interaction by diabetes type.

ESM Table 7. Associations between biomarkers of inflammation and changes in depressive symptoms (model 2).

Biomarkers	Total		T1D		T2D		$p_{interaction}$
	β	*p*	β	*p*	β	*p*	
ADA	0.009	0.824	-0.082	0.106	**0.194**	**0.005**	**0.003**
Axin-1	0.030	0.449	-0.043	0.425	**0.129**	**0.032**	**0.033**
Caspase-8	0.027	0.509	-0.027	0.612	0.130	0.053	0.054
CCL4	-0.051	0.201	**-0.108**	**0.035**	0.051	0.446	**0.026**
CCL19	-0.002	0.965	-0.082	0.141	0.103	0.108	**0.029**
CCL20	-0.049	0.216	**-0.143**	**0.010**	0.057	0.345	**0.011**
CCL23	0.007	0.852	-0.039	0.461	0.077	0.204	0.182
CCL25	0.001	0.979	-0.051	0.334	0.078	0.243	0.204
CCL28	-0.008	0.842	-0.029	0.603	0.017	0.806	0.755
CD5	-0.041	0.310	**-0.142**	**0.014**	0.069	0.237	**0.014**
CD6	-0.063	0.110	**-0.131**	**0.010**	0.059	0.374	**0.023**
CD8A	0.015	0.713	-0.062	0.248	**0.123**	**0.046**	**0.019**
CD40	-0.018	0.655	-0.099	0.063	**0.145**	**0.034**	**0.016**
CD244	-0.035	0.362	**-0.096**	**0.042**	0.119	0.112	**0.017**
CDCP1	-0.018	0.701	**-0.189**	**0.003**	**0.243**	**0.001**	**<0.001**
CSF-1	0.012	0.766	-0.072	0.173	**0.211**	**0.006**	**0.004**
CST5	0.027	0.506	0.001	0.981	0.077	0.223	0.487
CX3CL1	-0.000	0.999	-0.066	0.190	**0.136**	**0.046**	**0.041**
CXCL1	0.010	0.803	-0.057	0.286	0.115	0.066	**0.040**
CXCL5	0.009	0.818	0.019	0.739	0.006	0.927	0.931
CXCL6	0.017	0.672	-0.013	0.803	0.064	0.308	0.289
CXCL9	0.023	0.584	-0.056	0.309	**0.149**	**0.024**	0.056
CXCL10	0.043	0.286	-0.069	0.203	**0.204**	**0.001**	**0.002**
CXCL11	-0.010	0.790	-0.055	0.277	0.062	0.342	0.179
DNER	-0.030	0.487	-0.089	0.135	0.035	0.605	0.216
EIF4EBP1	0.015	0.714	-0.028	0.595	0.089	0.231	0.208
EN-RAGE	-0.005	0.902	-0.035	0.491	0.065	0.322	0.192
Eotaxin	0.018	0.670	-0.085	0.127	**0.205**	**0.004**	**0.005**
FGF-5	-0.053	0.187	-0.075	0.138	0.009	0.900	0.563
FGF-19	-0.044	0.296	-0.075	0.187	-0.001	0.991	0.367
FGF-21	-0.005	0.922	-0.072	0.243	**0.213**	**0.026**	**0.017**
Flt3L	0.000	0.991	-0.088	0.111	**0.146**	**0.032**	**0.016**
GDNF	0.025	0.538	-0.025	0.626	0.113	0.103	0.210
HGF	-0.030	0.473	-0.082	0.116	0.083	0.265	0.056
IFNγ	0.025	0.523	-0.057	0.323	0.104	0.062	0.066
IL-2RB	0.066	0.085	0.019	0.706	**0.135**	**0.028**	0.179
IL-6	0.026	0.588	-0.049	0.460	0.106	0.158	0.124
IL-7	0.044	0.262	0.000	0.996	0.111	0.062	0.139
IL-8	0.004	0.926	**-0.116**	**0.045**	**0.126**	**0.037**	**0.004**
IL-10	0.004	0.928	-0.111	0.053	**0.127**	**0.021**	**0.002**

IL-10RA	-0.013	0.745	-0.026	0.596	0.002	0.982	0.692
IL-10RB	-0.067	0.097	**-0.152**	**0.003**	0.130	0.076	**0.003**
IL-12B	0.014	0.728	-0.041	0.454	0.108	0.105	0.223
IL-15RA	-0.017	0.672	**-0.149**	**0.009**	**0.157**	**0.011**	**0.002**
IL-17A	0.009	0.818	-0.023	0.671	0.038	0.505	0.462
IL-17C	-0.071	0.067	**-0.100**	**0.046**	-0.050	0.453	0.522
IL-18	0.010	0.813	-0.052	0.348	0.104	0.110	0.062
IL-18R1	0.008	0.854	-0.082	0.161	**0.152**	**0.023**	**0.010**
LAP TGFβ1	-0.012	0.757	-0.039	0.415	0.056	0.440	0.251
LIF-R	-0.040	0.311	**-0.126**	**0.016**	0.093	0.155	**0.024**
MCP-1	0.015	0.705	-0.106	0.051	**0.193**	**0.002**	**<0.001**
MCP-2	0.015	0.701	-0.027	0.605	0.087	0.153	0.089
MCP-3	0.027	0.552	-0.074	0.206	**0.209**	**0.004**	**0.003**
MCP-4	0.011	0.783	-0.037	0.475	0.118	0.084	0.120
MIP-1α	-0.052	0.214	**-0.163**	**0.002**	**0.152**	**0.033**	**<0.001**
MMP-1	-0.018	0.635	0.011	0.835	-0.062	0.300	0.392
MMP-10	0.049	0.209	-0.001	0.989	**0.152**	**0.031**	0.133
NT-3	-0.041	0.309	-0.082	0.097	0.077	0.324	0.073
OPG	0.040	0.348	-0.063	0.279	**0.196**	**0.002**	**0.017**
OSM	0.023	0.562	-0.009	0.865	0.098	0.122	0.134
PD-L1	-0.005	0.889	**-0.166**	**0.004**	**0.154**	**0.005**	**<0.001**
SCF	0.019	0.656	0.054	0.389	0.001	0.982	0.339
SIRT2	0.016	0.682	-0.060	0.234	**0.162**	**0.014**	**0.006**
SLAMF1	-0.057	0.168	**-0.158**	**0.006**	0.048	0.432	**0.023**
ST1A1	-0.009	0.815	-0.053	0.294	0.084	0.208	0.062
STAMBP	0.035	0.372	-0.020	0.693	**0.128**	**0.048**	0.060
TGFα	0.001	0.986	-0.025	0.626	0.072	0.261	0.197
TNFRSF9	-0.024	0.543	-0.094	0.099	0.056	0.332	0.115
TNFSF14	0.008	0.831	-0.023	0.645	0.087	0.185	0.107
TNFα	0.058	0.151	-0.013	0.824	**0.126**	**0.027**	0.090
TNFβ	0.044	0.269	0.003	0.965	0.067	0.240	0.452
TRAIL	-0.054	0.165	-0.090	0.074	-0.003	0.967	0.245
TRANCE	-0.090	0.027	-0.046	0.394	**-0.175**	**0.009**	0.242
TWEAK	-0.008	0.852	0.004	0.945	-0.037	0.581	0.645
uPA	-0.021	0.591	**-0.117**	**0.026**	0.118	0.080	**0.012**
VEGF-A	-0.005	0.900	-0.036	0.483	0.065	0.333	0.198

Results are from model 2 adjusted for age, sex, study cohort, intervention/control group, baseline CES-D score, body mass index, HbA1c, diabetes duration, total cholesterol, triglycerides, use of lipid-lowering drugs, use of non-steroidal anti-

inflammatory drugs, use of antithrombotic medication and use of antidepressant medication. $p_{interaction}$ refers to interaction by diabetes type.

ESM Table 8. Associations between biomarkers of inflammation and changes in depressive symptoms (model 3)

**Biomarkers**	**Total**		**T1D**		**T2D**		$p_{interaction}$
	**β**	***p***	**β**	***p***	**β**	***p***	
ADA	0.009	0.820	-0.078	0.126	**0.199**	**0.004**	**0.003**
Axin-1	0.030	0.444	-0.040	0.452	**0.128**	**0.034**	**0.033**
Caspase-8	0.027	0.502	-0.024	0.653	0.128	0.057	0.054
CCL4	-0.050	0.203	**-0.106**	**0.038**	0.052	0.437	**0.027**
CCL19	-0.001	0.973	-0.080	0.151	0.101	0.116	**0.029**
CCL20	-0.049	0.218	**-0.141**	**0.012**	0.059	0.330	**0.011**
CCL23	0.008	0.839	-0.036	0.498	0.074	0.221	0.181
CCL25	0.002	0.969	-0.048	0.367	0.076	0.257	0.203
CCL28	-0.008	0.843	-0.026	0.641	0.023	0.738	0.772
CD5	-0.041	0.318	**-0.140**	**0.019**	0.065	0.264	**0.014**
CD6	-0.062	0.112	**-0.129**	**0.011**	0.058	0.382	**0.023**
CD8A	0.014	0.716	-0.060	0.267	**0.131**	**0.034**	**0.019**
CD40	-0.017	0.671	-0.095	0.080	**0.142**	**0.038**	**0.016**
CD244	-0.035	0.364	**-0.094**	**0.046**	0.123	0.102	**0.017**
CDCP1	-0.020	0.694	**-0.189**	**0.003**	**0.255**	**0.001**	**<0.001**
CSF-1	0.013	0.752	-0.068	0.206	**0.212**	**0.005**	**0.005**
CST5	0.028	0.497	0.008	0.880	0.078	0.219	0.499
CX3CL1	0.001	0.983	-0.062	0.233	**0.135**	**0.047**	**0.042**
CXCL1	0.010	0.796	-0.054	0.311	0.115	0.068	**0.041**
CXCL5	0.010	0.817	0.017	0.758	0.003	0.962	0.929
CXCL6	0.017	0.666	-0.010	0.844	0.064	0.305	0.294
CXCL9	0.023	0.575	-0.052	0.354	**0.149**	**0.025**	0.056
CXCL10	0.043	0.288	-0.069	0.208	**0.209**	**0.001**	**0.002**
CXCL11	-0.011	0.782	-0.055	0.281	0.069	0.291	0.185
DNER	-0.030	0.484	-0.087	0.142	0.038	0.574	0.221
EIF4EBP1	0.015	0.707	-0.024	0.643	0.088	0.236	0.209
EN-RAGE	-0.004	0.911	-0.034	0.504	0.061	0.356	0.185
Eotaxin	0.018	0.667	-0.082	0.148	**0.207**	**0.004**	**0.005**
FGF-5	-0.053	0.189	-0.073	0.154	0.012	0.868	0.568
FGF-19	-0.044	0.296	-0.073	0.199	0.001	0.986	0.372
FGF-21	-0.004	0.933	-0.070	0.262	**0.212**	**0.027**	**0.016**
Flt3L	0.0004	0.991	-0.087	0.116	**0.149**	**0.029**	**0.017**
GDNF	0.024	0.536	-0.022	0.671	0.118	0.090	**0.214**
HGF	-0.030	0.476	-0.080	0.128	0.085	0.258	0.057
IFNγ	0.025	0.523	-0.055	0.350	0.105	0.059	0.067
IL-2RB	0.066	0.087	0.017	0.741	**0.142**	**0.022**	0.180
IL-6	0.027	0.575	-0.044	0.508	0.103	0.170	0.122
IL-7	0.044	0.265	-0.001	0.981	0.115	0.056	0.140

IL-8	0.004	0.923	-0.113	0.056	**0.132**	**0.030**	**0.004**
IL-10	0.004	0.920	-0.107	0.065	**0.131**	**0.018**	**0.003**
IL-10RA	-0.013	0.743	-0.024	0.631	0.007	0.923	0.707
IL-10RB	-0.068	0.099	**-0.151**	**0.004**	0.128	0.081	**0.003**
IL-12B	0.015	0.713	-0.035	0.522	0.107	0.108	0.225
IL-15RA	-0.016	0.687	**-0.146**	**0.012**	**0.155**	**0.012**	**0.002**
IL-17A	0.009	0.813	-0.018	0.753	0.039	0.489	0.471
IL-17C	-0.071	0.068	-0.097	0.053	-0.050	0.454	0.528
IL-18	0.010	0.814	-0.050	0.371	0.108	0.098	0.063
IL-18R1	0.008	0.847	-0.080	0.176	**0.153**	**0.022**	**0.010**
LAP TGFβ1	-0.012	0.755	-0.039	0.411	0.059	0.418	0.255
LIF-R	-0.040	0.312	**-0.124**	**0.019**	0.100	0.130	**0.024**
MCP-1	0.016	0.698	-0.103	0.060	**0.193**	**0.002**	**<0.001**
MCP-2	0.015	0.698	-0.024	0.651	0.089	0.147	0.092
MCP-3	0.026	0.550	-0.072	0.221	**0.211**	**0.004**	**0.004**
MCP-4	0.012	0.774	-0.034	0.517	0.118	0.086	0.122
MIP-1α	-0.052	0.217	**-0.160**	**0.003**	**0.156**	**0.029**	**<0.001**
MMP-1	-0.018	0.644	0.014	0.795	-0.066	0.272	0.395
MMP-10	0.050	0.204	0.003	0.960	**0.152**	**0.031**	0.133
NT-3	-0.041	0.311	-0.081	0.104	0.080	0.310	0.075
OPG	0.040	0.347	-0.061	0.294	**0.201**	**0.002**	**0.018**
OSM	0.024	0.553	-0.008	0.885	0.095	0.139	0.131
PD-L1	-0.005	0.904	**-0.164**	**0.005**	**0.153**	**0.006**	**<0.001**
SCF	0.020	0.638	0.059	0.347	-0.005	0.939	0.343
SIRT2	0.016	0.678	-0.058	0.246	**0.163**	**0.014**	**0.006**
SLAMF1	-0.057	0.170	**-0.155**	**0.008**	0.051	0.406	**0.024**
ST1A1	-0.009	0.816	-0.054	0.286	0.083	0.216	0.062
STAMBP	0.035	0.367	-0.018	0.735	**0.128**	**0.047**	0.061
TGFα	0.001	0.976	-0.023	0.653	0.069	0.286	0.195
TNFRSF9	-0.024	0.557	-0.090	0.129	0.052	0.365	0.116
TNFSF14	0.009	0.826	-0.021	0.675	0.085	0.193	0.108
TNFα	0.058	0.149	-0.006	0.916	**0.130**	**0.023**	0.094
TNFβ	0.044	0.267	0.004	0.945	0.067	0.241	0.456
TRAIL	-0.054	0.168	-0.087	0.087	-0.008	0.903	0.245
TRANCE	**-0.089**	**0.028**	-0.043	0.423	**-0.180**	**0.007**	0.242
TWEAK	-0.008	0.845	0.005	0.927	-0.032	0.626	0.630
uPA	-0.022	0.587	**-0.116**	**0.027**	0.122	0.072	**0.012**
VEGF-A	-0.004	0.912	-0.033	0.518	0.061	0.373	0.194

Results are from model 3 adjusted for age, sex, study cohort, intervention/control group, baseline CES-D score, body mass index, HbA1c, diabetes duration, total

cholesterol, triglycerides, use of lipid-lowering drugs, use of non-steroidal anti-inflammatory drugs, use of antithrombotic medication, use of antidepressant medication and number of diabetes-related comorbidities. $p_{interaction}$ refers to interaction by diabetes type.

ESM Table 9. Associations between biomarkers of inflammation and changes in cognitive-affective symptoms (model 3)

Biomarkers	Total		T1D		T2D		$p_{interaction}$
	β	*p*	β	*p*	β	*p*	
ADA	0.000	0.996	-0.079	0.167	**0.196**	**0.013**	**0.007**
Axin-1	0.060	0.179	-0.033	0.585	**0.183**	**0.006**	**0.015**
Caspase-8	0.043	0.354	-0.029	0.628	**0.163**	**0.030**	0.056
CCL4	-0.059	0.194	**-0.123**	**0.032**	0.059	0.432	**0.042**
CCL19	-0.011	0.808	-0.091	0.148	0.114	0.117	**0.034**
CCL20	-0.049	0.275	-0.091	0.146	0.014	0.837	0.341
CCL23	0.027	0.541	0.006	0.918	0.066	0.332	0.664
CCL25	0.048	0.290	0.011	0.846	0.123	0.099	0.415
CCL28	0.034	0.471	-0.020	0.741	0.110	0.147	0.353
CD5	-0.016	0.723	-0.127	0.059	0.092	0.158	**0.014**
CD6	-0.059	0.186	**-0.138**	**0.015**	0.078	0.294	**0.009**
CD8A	0.045	0.317	-0.019	0.749	**0.159**	**0.023**	0.053
CD40	-0.005	0.922	-0.099	0.106	**0.195**	**0.011**	**0.007**
CD244	-0.004	0.928	-0.069	0.193	**0.191**	**0.021**	**0.012**
CDCP1	-0.014	0.794	**-0.178**	**0.013**	**0.264**	**0.002**	**0.002**
CSF-1	0.022	0.643	-0.073	0.229	**0.268**	**0.002**	**0.002**
CST5	0.027	0.564	-0.008	0.898	0.069	0.331	0.635
CX3CL1	-0.007	0.876	-0.083	0.151	0.147	0.056	**0.027**
CXCL1	0.006	0.893	-0.030	0.620	0.071	0.314	0.259
CXCL5	0.061	0.191	0.076	0.223	0.045	0.532	1.000
CXCL6	0.029	0.511	0.024	0.683	0.056	0.429	0.572
CXCL9	0.055	0.246	-0.002	0.976	**0.169**	**0.024**	0.322
CXCL10	0.054	0.235	-0.078	0.204	**0.258**	**0.000**	**0.001**
CXCL11	-0.005	0.911	-0.054	0.346	0.097	0.187	0.208
DNER	0.016	0.749	-0.103	0.119	**0.175**	**0.019**	**0.014**
EIF4EBP1	0.022	0.635	0.001	0.983	0.068	0.418	0.554
EN-RAGE	0.028	0.529	-0.013	0.816	0.093	0.200	0.235
Eotaxin	0.054	0.264	-0.015	0.813	**0.198**	**0.015**	0.120
FGF-5	0.006	0.901	-0.025	0.666	0.101	0.232	0.433
FGF-19	0.006	0.905	-0.022	0.733	0.053	0.483	0.521
FGF-21	0.008	0.881	-0.059	0.394	**0.241**	**0.025**	0.068
Flt3L	0.018	0.706	-0.058	0.348	**0.151**	**0.049**	0.156
GDNF	0.033	0.467	-0.042	0.462	**0.193**	**0.013**	0.066
HGF	-0.041	0.385	-0.068	0.247	0.044	0.605	0.209
IFNγ	0.045	0.319	-0.013	0.843	0.121	0.055	0.300
IL-2RB	0.072	0.098	0.023	0.687	**0.148**	**0.034**	0.264
IL-6	0.055	0.319	0.013	0.862	0.113	0.177	0.353
IL-7	**0.090**	**0.042**	0.014	0.818	**0.186**	**0.005**	**0.046**

IL-8	0.021	0.653	-0.093	0.162	**0.171**	**0.013**	**0.016**
IL-10	-0.010	0.824	-0.082	0.209	0.084	0.180	0.095
IL-10RA	-0.028	0.527	-0.017	0.755	-0.015	0.846	0.856
IL-10RB	-0.036	0.435	-0.115	0.052	0.141	0.087	**0.014**
IL-12B	0.032	0.493	-0.039	0.525	**0.169**	**0.024**	0.067
IL-15RA	0.002	0.965	**-0.152**	**0.020**	**0.222**	**0.001**	**0.001**
IL-17A	0.020	0.652	-0.002	0.974	0.037	0.562	0.671
IL-17C	-0.083	0.058	-0.099	0.080	-0.076	0.304	0.817
IL-18	0.037	0.424	-0.011	0.856	0.138	0.061	0.198
IL-18R1	0.013	0.790	-0.087	0.189	**0.172**	**0.022**	**0.007**
LAP TGFβ1	-0.013	0.764	-0.084	0.116	0.145	0.077	**0.018**
LIF-R	-0.021	0.634	**-0.117**	**0.049**	0.132	0.071	**0.036**
MCP-1	0.008	0.869	-0.072	0.239	0.139	0.050	**0.040**
MCP-2	0.057	0.199	0.002	0.969	**0.144**	**0.036**	0.104
MCP-3	0.038	0.452	-0.055	0.405	**0.221**	**0.007**	**0.011**
MCP-4	0.034	0.466	0.011	0.857	0.105	0.177	0.421
MIP-1α	-0.037	0.433	**-0.138**	**0.023**	**0.167**	**0.037**	**0.004**
MMP-1	0.043	0.333	0.079	0.183	-0.030	0.653	0.259
MMP-10	0.069	0.123	0.029	0.605	**0.171**	**0.031**	0.399
NT-3	-0.041	0.368	-0.093	0.093	0.120	0.172	**0.039**
OPG	0.032	0.507	-0.116	0.075	**0.246**	**0.001**	**0.004**
OSM	0.000	0.992	-0.006	0.916	0.030	0.682	0.424
PD-L1	-0.019	0.679	**-0.150**	**0.022**	**0.130**	**0.038**	**0.002**
SCF	0.089	0.066	**0.160**	**0.022**	0.020	0.765	0.198
SIRT2	0.033	0.460	-0.037	0.512	**0.168**	**0.023**	**0.027**
SLAMF1	-0.047	0.309	**-0.144**	**0.027**	0.070	0.309	**0.040**
ST1A1	-0.022	0.625	-0.042	0.453	0.029	0.697	0.434
STAMBP	0.046	0.308	-0.019	0.746	**0.164**	**0.023**	**0.048**
TGFα	-0.023	0.615	-0.024	0.676	0.011	0.876	0.466
TNFRSF9	0.011	0.811	-0.056	0.398	0.092	0.152	0.153
TNFSF14	-0.017	0.696	-0.032	0.567	0.038	0.605	0.254
TNFα	0.073	0.111	0.053	0.435	0.104	0.107	0.570
TNFβ	0.032	0.488	-0.011	0.865	0.061	0.336	0.352
TRAIL	-0.036	0.422	-0.066	0.247	0.014	0.853	0.289
TRANCE	-0.044	0.345	0.018	0.770	-0.148	0.051	0.302
TWEAK	0.030	0.528	0.007	0.914	0.041	0.582	0.610
uPA	-0.018	0.689	**-0.123**	**0.036**	0.143	0.060	**0.016**
VEGF-A	0.012	0.790	-0.003	0.954	0.051	0.500	0.417

Results are from model 3 adjusted for age, sex, study cohort, intervention/control group, baseline CES-D score, body mass index, HbA1c, diabetes duration, total

cholesterol, triglycerides, use of lipid-lowering drugs, use of non-steroidal anti-inflammatory drugs, use of antithrombotic medication, use of antidepressant medication and number of diabetes-related comorbidities. $p_{interaction}$ refers to interaction by diabetes type.

ESM Table 10. Associations between biomarkers of inflammation and changes in somatic symptoms (model 3)

Biomarkers	Total		T1D		T2D		$p_{interaction}$
	β	*p*	β	*p*	β	*p*	
ADA	0.008	0.852	-0.073	0.199	**0.186**	**0.022**	**0.010**
Axin-1	0.028	0.534	-0.054	0.369	0.135	0.052	0.052
Caspase-8	0.024	0.612	-0.033	0.578	0.122	0.117	0.113
CCL4	-0.081	0.075	**-0.154**	**0.007**	0.045	0.561	**0.012**
CCL19	-0.044	0.351	**-0.140**	**0.025**	0.091	0.226	**0.009**
CCL20	-0.036	0.431	-0.081	0.193	-0.002	0.976	0.353
CCL23	0.002	0.973	-0.014	0.816	0.025	0.723	0.693
CCL25	0.022	0.623	-0.039	0.503	0.087	0.260	0.335
CCL28	-0.075	0.110	-0.117	0.055	-0.009	0.907	0.407
CD5	-0.052	0.262	**-0.185**	**0.006**	0.085	0.206	**0.004**
CD6	-0.072	0.107	**-0.171**	**0.002**	0.119	0.117	**0.002**
CD8A	0.012	0.800	-0.060	0.315	0.121	0.093	**0.045**
CD40	-0.039	0.408	-0.109	0.072	0.082	0.306	0.087
CD244	-0.048	0.278	**-0.118**	**0.024**	0.149	0.084	**0.009**
CDCP1	-0.010	0.860	-0.140	0.051	**0.192**	**0.031**	**0.012**
CSF-1	-0.021	0.661	-0.105	0.078	0.168	0.058	**0.008**
CST5	0.007	0.882	-0.008	0.892	0.031	0.676	0.894
CX3CL1	-0.055	0.223	**-0.122**	**0.034**	0.054	0.497	0.165
CXCL1	0.020	0.668	-0.047	0.433	0.113	0.121	**0.043**
CXCL5	0.028	0.548	0.075	0.230	-0.019	0.800	0.635
CXCL6	0.036	0.422	-0.014	0.818	0.100	0.171	0.106
CXCL9	-0.005	0.912	-0.077	0.220	0.098	0.207	0.247
CXCL10	-0.008	0.868	-0.088	0.150	0.100	0.161	0.067
CXCL11	-0.043	0.343	-0.088	0.119	0.023	0.761	0.350
DNER	-0.072	0.144	**-0.163**	**0.013**	0.040	0.603	0.095
EIF4EBP1	-0.022	0.638	-0.054	0.350	0.018	0.837	0.514
EN-RAGE	0.028	0.532	-0.033	0.561	0.138	0.065	0.053
Eotaxin	0.010	0.838	-0.070	0.265	0.164	0.051	0.059
FGF-5	0.030	0.515	0.024	0.674	0.052	0.547	0.966
FGF-19	-0.040	0.406	-0.096	0.133	0.033	0.668	0.266
FGF-21	0.029	0.608	-0.017	0.804	0.206	0.063	0.062
Flt3L	0.014	0.769	-0.085	0.169	**0.161**	**0.042**	**0.033**
GDNF	-0.012	0.786	-0.084	0.139	0.113	0.164	0.154
HGF	-0.045	0.350	-0.112	0.057	0.115	0.185	**0.013**
IFNγ	0.007	0.873	-0.099	0.126	0.110	0.089	0.057
IL-2RB	0.019	0.662	-0.048	0.401	0.110	0.129	0.112
IL-6	0.012	0.829	-0.101	0.174	0.145	0.095	**0.027**
IL-7	0.020	0.653	**-0.118**	**0.024**	0.034	0.622	0.785

IL-8	-0.014	0.760	-0.123	0.062	0.076	0.287	**0.043**
IL-10	0.002	0.971	**-0.153**	**0.018**	**0.166**	**0.010**	**0.001**
IL-10RA	0.045	0.312	0.041	0.462	0.087	0.277	0.516
IL-10RB	-0.049	0.297	-0.112	0.057	0.086	0.314	**0.034**
IL-12B	0.019	0.692	-0.062	0.312	**0.166**	**0.032**	**0.043**
IL-15RA	-0.025	0.601	**-0.145**	**0.025**	0.129	0.072	**0.016**
IL-17A	0.050	0.263	0.000	0.997	0.104	0.114	0.265
IL-17C	-0.078	0.079	-0.094	0.093	-0.084	0.269	0.992
IL-18	0.016	0.728	-0.027	0.657	0.095	0.213	0.192
IL-18R1	0.018	0.718	-0.061	0.357	0.130	0.097	**0.049**
LAP TGFβ1	-0.015	0.742	-0.040	0.448	0.054	0.526	0.380
LIF-R	-0.027	0.555	**-0.147**	**0.013**	**0.156**	**0.038**	**0.007**
MCP-1	-0.008	0.864	-0.104	0.088	0.128	0.082	**0.013**
MCP-2	-0.002	0.961	-0.037	0.532	0.042	0.553	0.310
MCP-3	0.034	0.508	-0.070	0.284	**0.203**	**0.017**	**0.005**
MCP-4	0.003	0.944	-0.042	0.472	0.106	0.183	0.114
MIP-1α	-0.077	0.109	**-0.188**	**0.002**	0.114	0.168	**0.002**
MMP-1	-0.014	0.750	-0.024	0.685	-0.005	0.938	0.785
MMP-10	0.039	0.385	-0.001	0.984	0.115	0.163	0.363
NT-3	-0.013	0.779	-0.078	0.159	**0.198**	**0.028**	**0.008**
OPG	0.023	0.636	-0.111	0.087	**0.216**	**0.004**	**0.006**
OSM	0.031	0.494	-0.011	0.846	0.134	0.071	**0.039**
PD-L1	-0.001	0.977	**-0.150**	**0.021**	**0.138**	**0.033**	**0.002**
SCF	0.011	0.826	0.052	0.460	-0.037	0.595	0.269
SIRT2	0.010	0.830	-0.065	0.249	0.146	0.056	**0.030**
SLAMF1	-0.022	0.642	**-0.133**	**0.039**	0.110	0.120	**0.014**
ST1A1	-0.001	0.975	-0.034	0.546	0.069	0.373	0.288
STAMBP	0.015	0.736	-0.037	0.519	0.092	0.221	0.166
TGFα	0.027	0.546	-0.016	0.775	0.143	0.055	**0.034**
TNFRSF9	-0.007	0.877	-0.106	0.106	0.097	0.147	**0.048**
TNFSF14	0.033	0.461	0.010	0.855	0.117	0.120	0.116
TNFα	0.085	0.067	-0.001	0.992	**0.169**	**0.011**	0.072
TNFβ	0.009	0.852	-0.127	0.056	0.120	0.064	**0.012**
TRAIL	-0.072	0.109	-0.097	0.089	-0.070	0.387	0.526
TRANCE	-0.043	0.352	0.004	0.943	-0.118	0.131	0.493
TWEAK	-0.013	0.785	-0.007	0.914	-0.037	0.633	0.814
uPA	-0.038	0.399	**-0.143**	**0.015**	0.098	0.214	**0.022**
VEGF-A	-0.003	0.950	-0.062	0.281	0.124	0.113	**0.028**

Results are from model 3 adjusted for age, sex, study cohort, intervention/control group, baseline CES-D score, body mass index, HbA1c, diabetes duration, total

cholesterol, triglycerides, use of lipid-lowering drugs, use of non-steroidal anti-inflammatory drugs, use of antithrombotic medication, use of antidepressant medication and number of diabetes-related comorbidities. $p_{interaction}$ refers to interaction by diabetes type.

ESM Table 11. Associations between biomarkers of inflammation and changes in anhedonia symptoms (model 3)

**Biomarkers**	**Total**		**T1D**		**T2D**		$p_{interaction}$
	**β**	***p***	**β**	***p***	**β**	***p***	
ADA	0.006	0.888	-0.087	0.132	**0.200**	**0.014**	**0.002**
Axin-1	0.016	0.727	-0.067	0.274	0.129	0.065	**0.037**
Caspase-8	0.048	0.307	-0.019	0.752	**0.180**	**0.020**	**0.036**
CCL4	-0.041	0.367	**-0.127**	**0.027**	0.114	0.141	**0.006**
CCL19	0.022	0.634	-0.031	0.625	0.104	0.167	0.164
CCL20	-0.037	0.411	-0.109	0.084	0.071	0.317	**0.040**
CCL23	0.021	0.638	-0.011	0.860	0.060	0.397	0.401
CCL25	0.022	0.626	-0.013	0.826	0.095	0.218	0.230
CCL28	-0.020	0.671	-0.019	0.761	-0.023	0.770	0.813
CD5	0.036	0.437	-0.066	0.332	**0.156**	**0.020**	**0.017**
CD6	-0.039	0.387	**-0.138**	**0.016**	0.149	0.051	**0.003**
CD8A	0.045	0.323	-0.025	0.680	**0.152**	**0.035**	**0.046**
CD40	0.037	0.427	-0.036	0.556	**0.211**	**0.008**	**0.019**
CD244	-0.022	0.615	**-0.111**	**0.038**	**0.203**	**0.018**	**0.002**
CDCP1	-0.013	0.811	-0.135	0.062	**0.194**	**0.030**	**0.009**
CSF-1	0.005	0.917	-0.074	0.224	**0.221**	**0.013**	**0.006**
CST5	0.031	0.503	0.051	0.418	0.015	0.835	0.783
CX3CL1	-0.014	0.750	-0.069	0.236	0.099	0.216	0.096
CXCL1	0.042	0.353	-0.010	0.865	0.133	0.070	0.170
CXCL5	-0.001	0.976	-0.003	0.961	0.003	0.965	0.960
CXCL6	0.048	0.294	0.019	0.754	0.099	0.176	0.319
CXCL9	0.016	0.732	-0.007	0.916	0.065	0.405	0.582
CXCL10	0.028	0.538	-0.019	0.765	0.106	0.139	0.224
CXCL11	0.030	0.500	0.007	0.902	0.096	0.209	0.440
DNER	-0.037	0.461	-0.081	0.225	0.005	0.952	0.405
EIF4EBP1	0.041	0.391	-0.012	0.841	0.151	0.079	0.074
EN-RAGE	0.013	0.776	-0.014	0.815	0.070	0.352	0.315
Eotaxin	0.063	0.194	0.022	0.724	0.156	0.065	0.270
FGF-5	-0.060	0.190	-0.047	0.413	-0.084	0.334	0.690
FGF-19	-0.028	0.562	-0.077	0.230	0.023	0.764	0.222
FGF-21	0.040	0.491	-0.018	0.799	**0.284**	**0.010**	**0.028**
Flt3L	0.008	0.872	-0.047	0.452	0.105	0.190	0.231
GDNF	0.044	0.334	-0.003	0.962	0.145	0.073	0.195
HGF	0.038	0.429	-0.009	0.873	**0.177**	**0.042**	0.064
IFNγ	0.029	0.528	0.052	0.431	0.021	0.743	0.673
IL-2RB	0.078	0.077	0.031	0.594	**0.161**	**0.026**	0.258
IL-6	0.028	0.617	0.031	0.678	0.029	0.740	0.834
IL-7	0.066	0.142	0.016	0.795	**0.137**	**0.049**	0.129

IL-8	0.039	0.406	-0.036	0.588	**0.140**	**0.049**	**0.048**
IL-10	0.005	0.906	-0.074	0.259	0.121	0.062	**0.024**
IL-10RA	-0.071	0.109	-0.098	0.079	0.003	0.970	0.289
IL-10RB	-0.048	0.309	**-0.143**	**0.016**	**0.194**	**0.023**	**0.001**
IL-12B	0.013	0.783	-0.016	0.800	0.075	0.333	0.598
IL-15RA	0.013	0.777	-0.079	0.230	**0.145**	**0.043**	**0.026**
IL-17A	-0.035	0.438	-0.053	0.398	-0.007	0.916	0.628
IL-17C	**-0.091**	**0.041**	-0.108	0.057	-0.069	0.368	0.596
IL-18	0.038	0.427	0.025	0.684	0.067	0.382	0.520
IL-18R1	0.057	0.247	-0.048	0.473	**0.241**	**0.002**	**0.002**
LAP TGFβ1	0.001	0.979	-0.028	0.603	0.067	0.429	0.319
LIF-R	0.000	0.993	-0.101	0.090	**0.171**	**0.024**	**0.010**
MCP-1	0.051	0.272	-0.020	0.742	**0.174**	**0.018**	0.053
MCP-2	0.014	0.760	-0.028	0.638	0.106	0.139	0.083
MCP-3	0.039	0.447	-0.036	0.591	**0.191**	**0.025**	**0.041**
MCP-4	0.024	0.603	-0.034	0.572	**0.161**	**0.043**	0.113
MIP-1α	-0.021	0.658	**-0.129**	**0.035**	**0.183**	**0.027**	**0.002**
MMP-1	-0.002	0.965	0.057	0.343	-0.075	0.284	0.195
MMP-10	0.064	0.158	0.048	0.398	0.117	0.155	0.629
NT-3	-0.036	0.445	-0.084	0.133	0.109	0.231	0.070
OPG	0.065	0.176	-0.036	0.580	**0.225**	**0.003**	**0.033**
OSM	0.053	0.245	0.031	0.596	0.137	0.067	0.205
PD-L1	-0.003	0.939	-0.108	0.101	0.111	0.089	**0.012**
SCF	-0.042	0.395	0.008	0.913	-0.085	0.226	0.222
SIRT2	0.028	0.534	-0.077	0.172	**0.232**	**0.002**	**0.001**
SLAMF1	0.003	0.950	-0.061	0.350	0.086	0.226	0.114
ST1A1	-0.001	0.984	-0.068	0.229	0.142	0.066	**0.022**
STAMBP	0.044	0.334	-0.042	0.470	**0.198**	**0.008**	**0.006**
TGFα	0.051	0.257	0.023	0.700	**0.151**	**0.044**	0.158
TNFRSF9	0.007	0.879	-0.040	0.546	0.076	0.254	0.232
TNFSF14	0.051	0.254	-0.003	0.962	**0.183**	**0.015**	**0.030**
TNFα	0.048	0.301	-0.033	0.633	**0.145**	**0.029**	**0.041**
TNFβ	0.031	0.500	-0.036	0.592	0.083	0.206	0.251
TRAIL	-0.039	0.392	-0.034	0.558	-0.042	0.601	0.960
TRANCE	**-0.102**	**0.030**	-0.051	0.396	**-0.186**	**0.018**	0.200
TWEAK	-0.007	0.884	-0.066	0.304	0.056	0.469	0.304
uPA	-0.010	0.828	-0.111	0.061	0.139	0.077	**0.022**
VEGF-A	0.018	0.698	-0.006	0.917	0.094	0.234	0.268

Results are from model 3 adjusted for age, sex, study cohort, intervention/control group, baseline CES-D score, body mass index, HbA1c, diabetes duration, total

cholesterol, triglycerides, use of lipid-lowering drugs, use of non-steroidal anti-inflammatory drugs, use of antithrombotic medication, use of antidepressant medication and number of diabetes-related comorbidities. $p_{interaction}$ refers to interaction by diabetes type.

ESM Figure 1. Histogram of correlation coefficients for pairwise coorrelations among 76 biomarkers of inflammation.

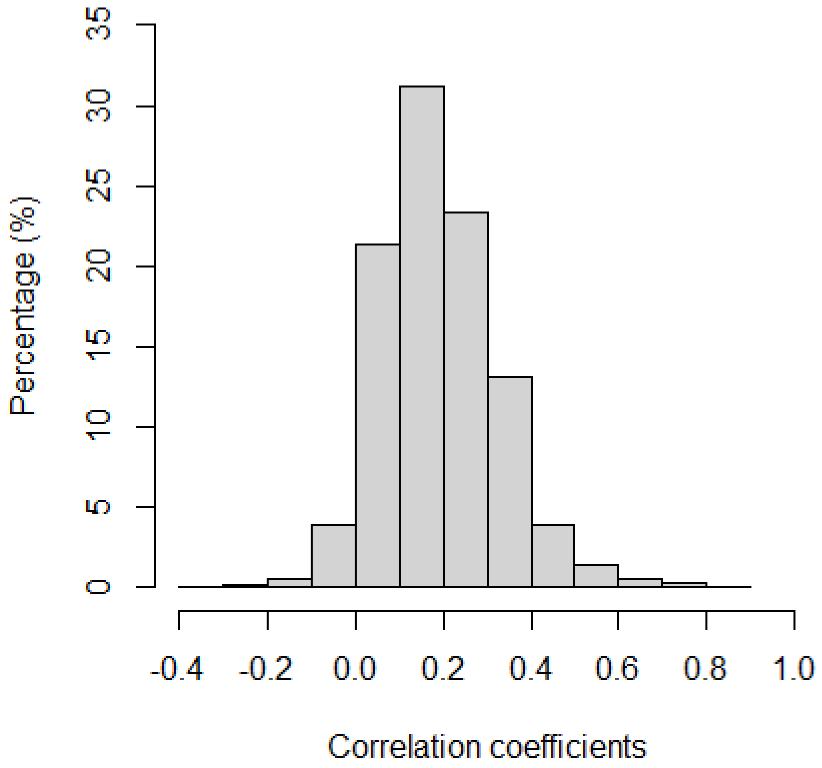

ESM Figure 2. Chord diagram of correlations between 76 biomarkers of inflammation and clinical characteristics of the study participants at baseline.

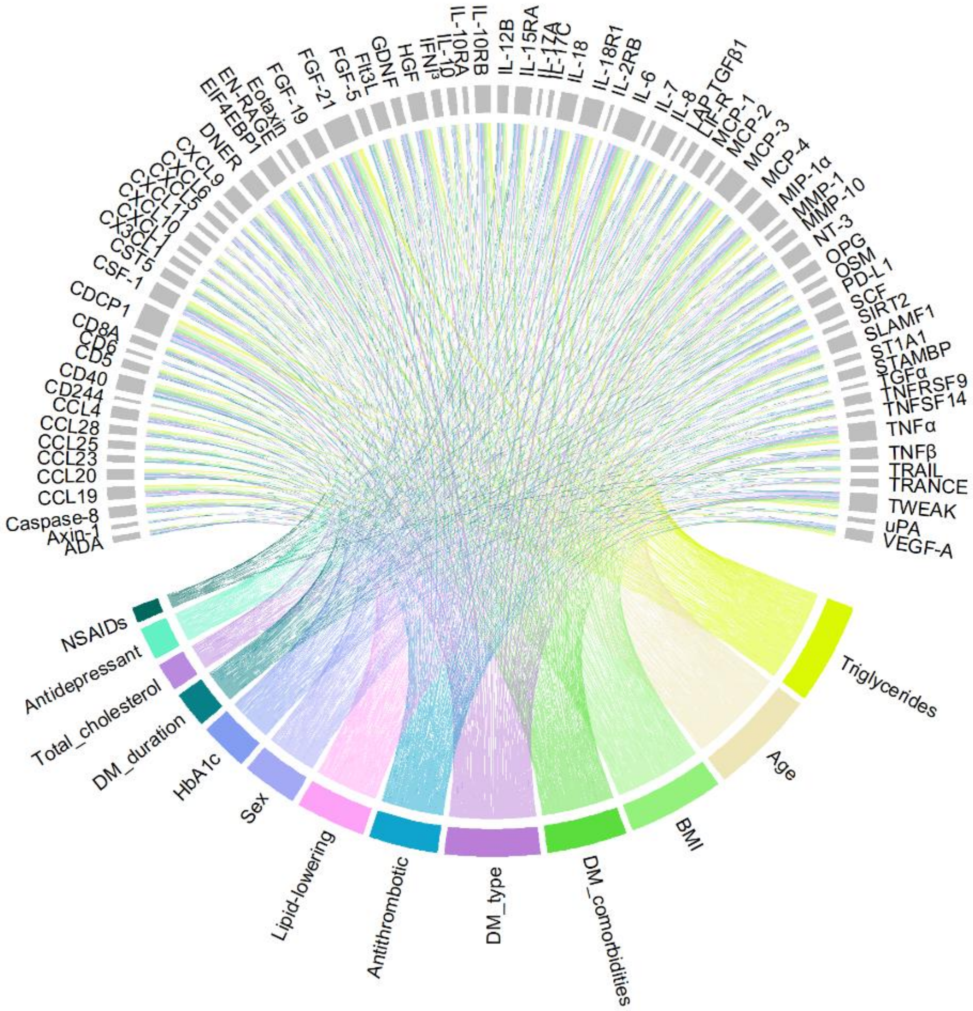

DM, diabetes mellitus; NSAIDs, non-steroidal anti-inflammatory drugs.

ESM Figure 3. Heat map of correlations between 76 biomarkers of inflammation and baseline characteristics of the study participants at baseline.

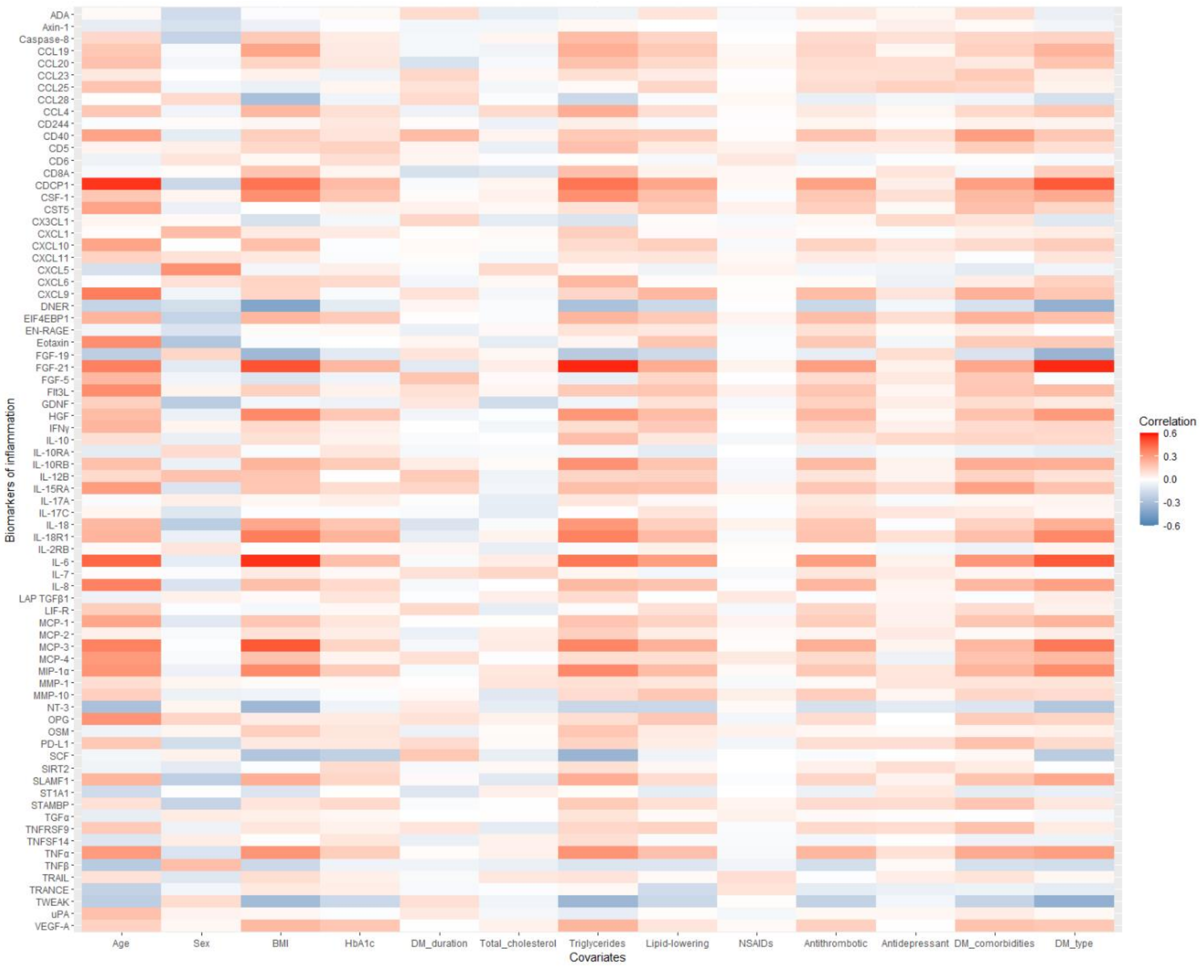

The diagram visualises all pairwise correlations between biomarkers of inflammation and clinical characteristics in the total study sample.

Categorical variables were labelled as follows: sex, 1=male, 2=female; use of ipid-lowering drugs, NSAIDs, antithrombotic drugs and antidepressant drugs, 0=no, 1=yes; DM_type, 1=type 1 diabetes, 2=type 2 diabetes.

DM, diabetes mellitus; NSAIDs, non-steroidal anti-inflammatory drugs.
